# Amyloid-beta modulates the association between neurofilament light chain and brain atrophy in Alzheimer’s disease

**DOI:** 10.1038/s41380-020-0818-1

**Published:** 2020-06-26

**Authors:** Min Su Kang, Arturo Aliaga Aliaga, Monica Shin, Sulantha Mathotaarachchi, Andrea L. Benedet, Tharick A. Pascoal, Joseph Therriault, Mira Chamoun, Melissa Savard, Gabriel A. Devenyi, Axel Mathieu, M. Mallar Chakravarty, Åsa Sandelius, Kaj Blennow, Henrik Zetterberg, Jean-Paul Soucy, A. Claudio Cuello, Gassan Massarweh, Serge Gauthier, Pedro Rosa-Neto, Alzheimer’s Disease Neuroimaging Initiative

**Affiliations:** 1 Translational Neuroimaging Laboratory, McGill University Research Centre for Studying in Aging, Montreal, QC Canada; 2 Cerebral Imaging Centre, Douglas Research Centre, Montreal, QC Canada; 3 McConnell Brain Imaging Centre, McGill University, Montreal, QC Canada; 4 Department of Psychiatry, McGill University, Montreal, QC Canada; 5 Department of Biomedical Engineering, McGill University, Montreal, QC Canada; 6 Department of Psychiatry and Neurochemistry, The Sahlgrenska Academy at the University of Gothenburg, Mölndal, Sweden; 7 Clinical Neurochemistry Laboratory, Sahlgrenska University Hospital, Mölndal, Sweden; 8 UK Dementia Research Institute at UCL, London, UK; 9 Department of Neurodegenerative Disease, UCL Institute of Neurology, Queen Square, London, UK; 10 Department of Pharmacology and Therapeutics, McGill University, Montreal, QC Canada

**Keywords:** Neuroscience, Molecular biology, Biomarkers, Diseases

## Abstract

Neurofilament light chain (NFL) measurement has been gaining strong support as a clinically useful neuronal injury biomarker for various neurodegenerative conditions. However, in Alzheimer’s disease (AD), its reflection on regional neuronal injury in the context of amyloid pathology remains unclear. This study included 83 cognitively normal (CN), 160 mild cognitive impairment (MCI), and 73 AD subjects who were further classified based on amyloid-beta (Aβ) status as positive or negative (Aβ+ vs Aβ−). In addition, 13 rats (5 wild type and 8 McGill-R-Thy1-APP transgenic (Tg)) were examined. In the clinical study, reduced precuneus/posterior cingulate cortex and hippocampal grey matter density were significantly associated with increased NFL concentrations in cerebrospinal fluid (CSF) or plasma in MCI Aβ+ and AD Aβ+. Moreover, AD Aβ+ showed a significant association between the reduced grey matter density in the AD-vulnerable regions and increased NFL concentrations in CSF or plasma. Congruently, Tg rats recapitulated and validated the association between CSF NFL and grey matter density in the parietotemporal cortex, entorhinal cortex, and hippocampus in the presence of amyloid pathology. In conclusion, reduced grey matter density and elevated NFL concentrations in CSF and plasma are associated in AD-vulnerable regions in the presence of amyloid positivity in the AD clinical spectrum and amyloid Tg rat model. These findings further support the NFL as a neuronal injury biomarker in the research framework of AD biomarker classification and for the evaluation of therapeutic efficacy in clinical trials.

## Introduction

Alzheimer’s disease (AD) is defined by the presence of amyloid and tau pathologies that lead to neuronal injury or neurodegeneration and cognitive decline. Although the exact aetiology of AD is still being debated, the most widely accepted amyloid cascade hypothesis rests on amyloid-beta (Aβ) as the initiating event leading to a cascade of AD pathophysiological processes [1]. This hypothesis has gained strong support based on the multitude of biomarker-based evidence suggesting that Aβ abnormality occurs decades before the onset of AD [2]. This evidence suggests that the Aβ pathology correlates only weakly with neuronal injury or neurodegeneration but its downstream tau pathology is more strongly associated with neurodegeneration and consequently cognitive decline [3]. As such, the new NIA-AA research framework A/T/(N) focuses on measurable neuropathologic biomarkers to define and track patients along the AD continuum for staging purposes [4]. Following this framework, A (aggregated Aβ) and T (aggregated tau) define AD and can be measured using positron emission tomography (PET) or cerebrospinal fluid (CSF) biomarkers, while *N* (neuronal injury or neurodegeneration) is not specific to AD but provides staging of AD pathophysiological processes and can be measured using magnetic resonance imaging (MRI), [^18^F]FDG-PET, and CSF total tau (T-tau) [4]. Effectively, the biomarkers of *N* allow predicting cognitive decline or conversion for clinical trial enrichment [5].

Recent studies suggest neurofilament light chain (NFL) as a putative biomarker for neuronal injury or neurodegeneration. NFL is a subunit of the neurofilament that plays an important role in axonal and dendritic branching and growth, and axonal integrity [6, 7]. Elevated NFL concentration in CSF has been associated not only with the axonal damage but also with neuronal injury, brain atrophy and disease severity in various neurological disorders, including AD, and in their respective animal models [8–16]. Importantly, it has already been demonstrated that CSF and plasma NFL levels are correlated to each other and are elevated in AD [17].

To maximize the utility of a novel biomarker and its proper use in AD research and therapeutic trials, it is imperative to compare it with other well-established biomarkers. In the case of NFL, this would facilitate its incorporation among the existing biomarkers [4]. Current evidence on the influence of Aβ pathology on NFL levels has been conflicting. Some previous studies suggest that NFL levels may be independent of Aβ pathology as there was no statistical difference between Aβ− and Aβ+ subjects in the clinical spectrum of sporadic AD [11, 18]. However, a recent study showed that CSF and serum NFL levels were increased in pre-symptomatic familial AD mutation carriers and the rate of increase in the serum NFL was able to discriminate the mutation carriers from non-carriers almost a decade before the expected onset of symptoms [19]. Furthermore, a recent study on the Alzheimer’s Disease Neuroimaging Initiative (ADNI) cohort showed that longitudinal increases in plasma NFL levels correlate with baseline CSF indices of brain amyloidosis [20]. Moreover, it was revealed that NFL concentration was negatively associated with grey matter volumes of a priori defined regions of interest (ROI) that are vulnerable in AD—hippocampus, precuneus, or temporal composite regions (entorhinal, inferior temporal, middle temporal, and fusiform cortex) [11, 17, 19]. However, the empirical evidence on the NFL levels association with reduced grey matter density at a voxel-based regional level in the context of Aβ pathology is largely missing. Although NFL elevation is an indicator of the presence of neurodegenerative processes, this fluid biomarker lacks the topographical information present in [^18^F]FDG-PET or MRI scans [21, 22]. This is an important limitation since plasma or CSF NFL levels might reflect disease-specific grey or white matter neurodegenerative processes or disease-independent healthy aging process [22–24]. Understanding the topographical correlates between NFL and neuronal injury or neurodegeneration in the context of Aβ pathology is crucial to interpret NFL levels as a novel fluid biomarker for neuronal injury or neurodegeneration in AD research and therapeutic trials.

In this study, we elucidate the contributing role of Aβ pathology on neuronal injury reflected by NFL concentration and grey matter density maps. In addition, we illustrate voxel-based correlates between NFL and grey matter density maps in humans and McGill-Thy1-APP transgenic (Tg) rat model [25]. This Tg model is ideal to study the effect Aβ alone as it progressively develops Aβ pathology comparable to AD Aβ pathology without the presence of tau pathology or minimal cell death [25–27]. The present study highlights that NFL concentration reflects neuronal injury processes in the regions that are vulnerable to AD in the presence of Aβ pathology.

## Materials and methods

### Subjects

#### Human dataset

Data used in the preparation of this article were obtained from the ADNI (adni.loni.usc.edu). The ADNI was launched in 2003 by the National Institute on Aging (NIA), the National Institute of Biomedical Imaging and Bioengineering (NIBIB), the Food and Drug Administration (FDA), private pharmaceutical companies and non-profit organizations, as a $60 million, 5-year public–private partnership. ADNI is the result of efforts of many co-investigators from a broad range of academic institutions and private corporations, and subjects have been recruited from over 50 sites across the USA and Canada. For up-to-date information, see www.adni-info.org.

The criteria for cognitively normal (CN) classification are mini-mental state examination (MMSE) scores between 24 and 30 (inclusive) without cognitive complaints or observable anomalies. The criteria for the diagnosis of mild cognitive impairment (MCI) included the presence of memory complaints, MMSE scores between 24 and 30 (inclusive), objective memory loss, Clinical Dementia rating (CDR) of 0.5, preserved activities of daily living, and absence of dementia. Mild AD dementia subjects had MMSE scores between 20 and 26 (inclusive), CDR of 0.5 or 1.0, and fulfilled the NINCDS/ADRDA criteria for probable AD.

#### Animal dataset

All procedures described here were performed in compliance with ethics protocols approved by McGill Animal Care Ethics Committee and following the Canadian Council on Animal Care guidelines. All rats were housed at the Douglas Mental Health University Institute animal facility on a 12/12 h light/darkness cycle with ad libidum access to food and water. A starting sample of 26 animals was included in this study based on the effect size estimated from the previous study [12]. Eleven were wild type (WT) animals and 15 were homozygous McGill-R-Thy1-APP Tg rats were randomly selected. All the animals were on Wistar background for the study. All the animal procedures were completed blinded to the genotype.

### Inclusion and exclusion criteria

#### Human

ADNI inclusion and exclusion criteria can be found at (www.adni-info.org). For our study, we included 83 CN, 160 MCI, and 73 AD individuals who have both CSF and plasma NFL, CSF Aβ_42_, CSF phosphorylated tau (p-tau), and structural MRI data available. For plasma NFL analysis, we identified two CN, eight MCI, and four AD subjects as outliers (two standard deviations away from the mean) who were excluded in the analysis.

#### Animals

All animal data points that overlap with any sign of brain tumour development or had a major surgery to remove tumour were excluded within the longitudinal data points (four WT and seven Tg). Last, all animals that were identified as an outlier (two standard deviations away from the mean) were excluded (two WT).

### Imaging acquisition and processing

#### Human

Details on the image acquisition parameters can be found here (http://adni.loni.usc.edu/methods). All T1-weighted MRIs were processed using an in-house processing pipeline and underwent non-uniformity correction [28], brain masking [29], normalization to the Montreal Neurological Institute (MNI) space using affine and nonlinear transformation with Advanced Normalization Tools (ANTs) registration tools [30, 31], and segmented using three-tissue priors in MNI space (grey matter, white matter, and CSF). Our in-house pipeline based on Medical Imaging NetCDF (MINC) toolkits (www.bic.mni.mcgill.ca/ServicesSoftware) generated Voxel-based morphometry (VBM) images representing grey matter density maps. In brief, a log Jacobian determinant was derived based on the nonlinear vector field from the previously mentioned pipeline. Then, it was transformed into a scalar, modulated with grey matter probability mask, and smoothed with 8 mm full-width at half-maximum (FWHM). Last, three ROIs from the MNI template atlas were used to replicate the previous findings in the literature: (1) temporal composite (Temp.Comp; bilateral middle and inferior temporal gyri, fusiform and entorhinal cortices, and hippocampi), (2) Entorhinal cortices, and (3) hippocampi [11, 17, 20].

#### Animals

All MRI images were acquired using Bruker 7T BioSpec 70/30 USR dedicated for small animal. All rats were under 1.5% isoflurane anaesthesia during the scan after a 5% anaesthesia induction.

All structural images were obtained at 15 months using the Bruker standard 3D-True Fast Imaging with Steady State Precession pulse sequence (3D-TruFISP). A root-mean-square image of eight phases acquisition was performed. Each angle acquisition was acquired in a field-of-view of 36 × 36 × 36 mm with a matrix of 180× 180× 180× of TE/TR of 2.5/5.0 ms with a flip angle of 30° and NEX of 2. The final image was an average of the 16 acquisitions with a final 250 µm isotropic resolution and a total acquisition time of 46 min scan.

All images were processed using MINC toolkits (www.bic.mni.mcgill.ca/ServicesSoftware). All individual MRI images were aligned with rigid body transformation, affine, and nonlinear transformation, and matched to generate an average population-based template based on Pydpiper [32].

Nonlinear vector field was used to create a log Jacobian determinant for deformation-based morphometry (DBM) representing local volume difference in each subject [32]. Considering rodent brain has white matter in limited regions, these local changes in the neocortex were considered grey matter changes. Finally, 1 mm FWHM smoothing was applied. Then DBM was transformed to a scalar as VBM values for ROI analysis. Last, three ROIs based on the Paxino and Watson atlas 6th edition were used: (1) temporal composite (Temp.Comp; bilateral temporal associative cortices and hippocampi), (2) Entorhinal cortices, and (3) hippocampi.

### Fluid collection and analysis

#### Human CSF

CSF collection, processing, and storage procedures have been described previously (www.adni-info.org). CSF Aβ_42_ and CSF p-tau concentrations were measured using the Elecsys on a cobas e 601 analyzer as described previously [33, 34]. CSF NFL concentration was measured using a commercially available enzyme-linked immunosorbent assay (NF-light; UmanDiagnostics) as described by the manufacturer. The measurements were performed by board-certified laboratory technicians, who were masked to clinical data, using one batch of reagents. Intrabatch coefficients of variation were below 10%. Amyloid positivity was classified based on the previously established CSF p-Tau/Aβ_42_ ratio cutoff threshold (CSF p-Tau/Aβ_42_ > 0.025) [34].

#### Human plasma NFL

Plasma NFL collection, processing, and storage procedures have been described previously (www.adni-info.org). In brief, plasma NFL concentration was measured using an in-house single-molecule array (Simo) method, as described previously in detail [35]. The measurements were performed by board-certified laboratory technicians, who were masked to clinical data, using one batch of reagents. For runs in this study, the coefficient of variations (CV) for the low (11.0 ng/L) quality control (QC) sample was 6.2% for intra-assay CV and 9.0% for inter-assay CV, while the corresponding values for the high (173.0 ng/L) QC sample were 4.9% for intra-assay CV and 7.2% for inter-assay CV.

#### Animals CSF

All animals were anesthetized with a 5% isoflurane induction for 5–8 min and placed in a stereotaxic apparatus. The CSF samples (100–150 µL) were collected via direct puncture through the cisterna magna at 10–18 months of age.

Rat CSF NFL concentration was determined using the in-house Simoa NFL assay which has been described in detail previously [16], with some modifications. Briefly, samples were diluted 100× with assay diluent and incubated for 35 min with paramagnetic carboxylated beads (Quanterix Corp, Boston, MA, USA) coated with a mouse anti-neurofilament light antibody (UD1, UmanDiagnostics, Umeå, Sweden) and a biotinylated mouse anti-neurofilament light antibody (UD2, UmanDiagnostics) in a Simoa HD-1 instrument (Quanterix). The bead-conjugated immunocomplex was thoroughly washed before incubation with streptavidin-conjugated β-galactosidase (Quanterix). Thereafter, the bead complex was washed and resorufin β-D-galactopyranoside (Quanterix) was added. The immunocomplex was applied to a multi-well array designed to enable imaging of every single bead. The average number of enzymes per bead (AEB) of samples was interpolated using the calibrator curve constructed by AEB measurements on bovine NFL (UmanDiagnostics) serially diluted in assay diluent. Samples were analyzed ‘blind’ and in duplicate using one batch of reagents. The average repeatability coefficient of variation of a sample with a mean concentration of 18,102 pg/mL was 8.5% and intermediate precision was 8.8%, and for a sample with a mean concentration of 9460 pg/mL, repeatability was 8.1% and intermediate precision was 11.5%. All samples analyzed were above the lower limit of quantification (LLoQ).

Rat CSF concentrations of Aβ_42_ and Aβ_40_ were measured using Quanterix kits according to kit instructions (Simoa Aβ_42_ 2.0 211 and Simoa Aβ_40_ 2.0 218, Quanterix) with a CSF dilution of 100× using the provided assay diluents. For Aβ_42_ CSF measurements, the average repeatability coefficient of variation of a sample with a mean concentration of 1650 pg/mL was 3.2% and intermediate precision was 9.2%, and for a sample with a mean concentration of 641 pg/mL, repeatability was 10.9% and intermediate precision was 16.6%. For Aβ_40_ CSF measurements, repeatability for a sample with a concentration 5160 pg/mL was 17.2%, and intermediate precision 17.2%, and for a sample with a concentration of 3975 pg/mL repeatability and intermediate precision was 7.3% and 11.8%, respectively. For both assays, a number of samples were below the assigned assay limit of quantification and assigned a concentration of half of the LLoQ.

#### Statistical analysis

Potential difference in the number of male and female in each group was tested based on a chi-square test for both humans and animals as well as APOEε4 status in humans. We also tested whether there were differences in age, education, and MMSE scores for humans based on a two-sided ANOVA. We tested if WT and Tg had any difference in age using an unpaired two-sided *t* test. We log transformed NFL in CSF and plasma, as well as CSF Aβ_42_ and p-tau.

In animals, linear mixed effect models with a random intercept were performed to measure longitudinal changes in CSF Aβ_42/40_ and CSF NFL log: (1) CSF Aβ_42/40_ ~ Age + (1|animals); (2) CSF NFL (log) ~ Age (WT or Tg) + (1|animals); (3) CSF NFL (log) ~ Age + Genotype + Age × Genotype + (1|animals). The association between CSF NFL and CSF Aβ_42/40_ was investigated based on a linear regression model. Weight was retained as a variable only in the model showing the association between CSF NFL and CSF Aβ_42/40_ as it was shown to have a significant effect.

In animals and humans, voxel-based linear regression models were performed to detect the group contrast in DBM and VBM, respectively. Furthermore, a voxel-based linear regression was performed between DBM and CSF NFL log at 15 months for animals, and VBM and CSF or plasma NFL levels for humans: animal model: CSF NFL log ~ DBM; human model: NFL (CSF or plasma) ~ VBM + covariates in each group as well as combining all groups with adjusting for the diagnosis. Similarly, we used those same models as the voxel-based models to replicate the previous findings using the Temp.Comp, entorhinal, and hippocampus ROIs with FDR correction for animals and humans [11, 17, 20]. In addition, we applied bootstrapping technique in the group that showed significant association between NFL and VBM, running the standardized ROI-based models 2000 times to compare the effect size between Aβ− vs Aβ+. All voxel-based and ROI-based analyses and linear regression models examining the grey matter density and NFL concentrations in humans were corrected for age, sex, APOEε4 status, education, CSF p-tau (log), and the difference in dates between the NFL measurements and MRI scan. Furthermore, additional CSF Aβ_42_:p-tau interaction term was tested to exclude the effect of p-tau and Aβ_42_ interaction when the term was significant.

All voxel-based analyses were conducted using VoxelStats and corrected for multiple comparison using random field theory with peak threshold at *p* = 0.05 and cluster threshold at *p* = 0.05 for rats and *p* = 0.005 for human [36]. All the statistical analyses were conducted using R 3.4.4.

## Results

### Demographic

Table 1 summarizes the demographic of the participants included in the study. There was no difference in age or education between groups. However, the number of males and females was different between CN Aβ+ vs MCI Aβ− and MCI Aβ− vs AD Aβ+. APOEε4 status was different between groups except for CN Aβ− vs MCI Aβ−, CN Aβ+ vs MCI Aβ+, and MCI Aβ+ vs AD Aβ+. Furthermore, average MMSE score was not different between CN Aβ− vs CN Aβ+ and MCI Aβ− vs MCI Aβ+, whereas all other comparisons showed significant differences.

**Table 1:** Demographics.

Groups	CN (*n* = 83)	CN Aβ− (*n* = 66)	CN Aβ+ (*n* = 17)	MCI (*n* = 160)	MCI Aβ− (*n* = 48)	MCI Aβ+ (*n* = 112)	AD Aβ+ (*n* = 73)
Age (years)	75.90 ± 5.47	75.65 ± 5.36	76.91 ± 5.95	74.61 ± 7.53	75.27 ± 7.99	74.32 ± 7.34	74.03 ± 7.64
Sex (M/F)	44/39	32/34	12/5	111/49	39/9^a,^**	72/40	42/31^c,^*
APOEε4 carrier (%)	17 (20.5%)	6 (9.1%)	11 (64.7%)^a,^***	82 (51.3%)	9 (23.1%)^b,^**	73 (65.2%)^a,^***^,c,^***	57 (78%)^a,^***^,b,^**^,c,^***
Education	15.77 ± 2.83	15.58 ± 2.73	16.53 ± 3.16	15.72 ± 3.03	15.90 ± 3.16	15.64 ± 2.99	14.95 ± 3.25
MMSE	28.99 ± 1.10	29.00 ± 1.12	28.94 ± 1.03	26.96 ± 1.79	27.33 ± 1.68^a,^***^,b,^**	26.79 ± 1.82^a,^***^,b,^***	23.56 ± 1.74^a,^***^,b,^***^,c,^***^,d,^***
Groups	WT (*n* = 5)	Tg (*n* = 8)					
Age (years)	10–18	10–18					
Sex (M/F)	3/2	6/2					
Weight (g)	583.7 ± 260.2	601.2 ± 161.8					

All values are indicated as mean ± standard deviation except for sex and APOEε4. The *p*-value indicates the value assessed with analyses of variance (ANOVA) among Aβ− and Aβ+ CN, MCI, and AD for each variable except for sex and APOEε4, where a contingency chi-square test was performed with false discovery rate multiple corrections.

*CN* cognitively normal, *MCI* mild cognitive impairment, *AD* Alzheimer’s disease, *Aβ*− amyloid-beta negative, *Aβ*+ amyloid-beta positive, *MMSE* mini-mental state examination.

Post-hoc Bonferroni analysis provided significant differences between groups: ^a^from CN Aβ−; ^b^CN Aβ+; ^c^MCI Aβ−; ^d^MCI Aβ+; *p < 0.05, **p < 0.005; ***p < 0.0001.

For the animal data, there was no difference in age, sex, or weight (Table 1).

### Longitudinal CSF biomarker changes and their association and grey matter changes in animals

**Fig. 1:**
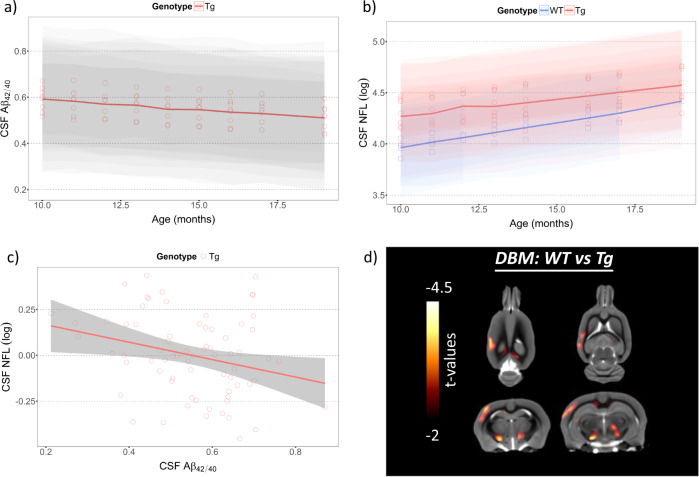
Natural progression in CSF biomarkers and grey matter density changes in control and Tg animals. **a, b** Longitudinal analyses on CSF Aβ_42/40_ and NFL concentrations, **c** an association between CSF Aβ_42/40_ and NFL concentration, and **d** voxel-based group contrast between WT and Tg in DBM with the *t*-statistic scale. The shades in the regression lines represent 95% confidence intervals of the model.

In McGill-Thy1-APP Tg rats only, a linear mixed effect analysis showed a significant longitudinal decline in CSF Aβ_42/40_ from 10 months to 18 months (*β* = −0.01, standard error (s.e) = 0.0045, *t*(63) = −2.09, *p* = 0.041) (Fig. 1a). On the other hand, the longitudinal CSF NFL concentration significantly increased in both WT (*β* = +0.05, s.e = 0.012, *t*(27) = 4.06, *p* = 0.0004) and Tg (*β* = +0.04, s.e = 0.006, *t*(64) = 5.63, *p* < 0.0001) (Fig. 1b). When we analyzed both groups into one model by including an interaction term between the group and time, the CSF NFL concentration was progressively increased over time (*β* = +0.05, s.e = 0.01, *t*(93)   4.38, *p* < 0.0001) while Tg had a significantly greater CSF NFL concentration compared to WT (*β* = +0.43, s.e = 0.2, *t*(90) = 2.14, *p* = 0.035) (Fig. 1b). However, the interaction between the genotype effect and longitudinal change in CSF NFL concentration was not significant. Similar to previous human findings, the significant inverse association between CSF NFL and CSF Aβ_42/40_ was observed when adjusted for weight (*β* = −0.5, s.e = 0.2, *t*(68) = −2.40, *p* = 0.02) (Fig. 1c).

Voxel-based group contrast in DBM at 15 months showed significant reduction in parietotemporal cortex (*t*(11) = 3.2, *p* = 0.008), bilateral dorsal hippocampi (*t*(11) = 2.3, *p* = 0.042), and striatum in Tg compared to WT with the greatest contrast seen in thalamus (*t*(11) = 5, *p* = 0.0004) (Fig. 1d and Supplementary Fig. 1 and Table 1).

### CSF NFL and DBM association is modulated by amyloid in AD-vulnerable regions in Tg

**Fig. 2:**
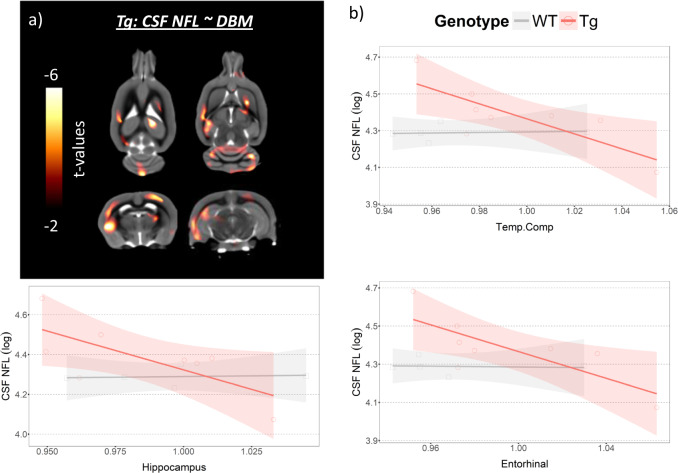
Increased CSF NFL concentrations are associated with reduced grey matter density in transgenic animals. **a** The association between CSF NFL concentration and DBM at 15 months of age in Tg with the *t*-statistic scale. **b** The association between CSF NFL concentration and ROIs from Temp.Comp, entorhinal, and hippocampus VBM in WT and Tg. The shades in the regression lines represent 95% confidence intervals of the model.

Increased CSF NFL concentration was significantly associated with reduced DBM only in Tg, encompassing the parietotemporal cortex, hippocampus, striatum, thalamus, and cerebellum in the voxel-based analysis. The greatest effect was observed in the parietotemporal cortex (*t*(6) = 13, *p* = 0.0001) and hippocampus (*t*(6) = 8, *p* = 0.0002) (Fig. 2a and Supplementary Fig. 2 and Table 2). The ROI-based linear regression analyses revealed significant negative associations between CSF NFL concentration and the Temp.Comp VBM (*β* = −4.09, s.e = 1.29, *t*(6) = −3.18, *p* = 0.037), entorhinal cortex (*β* = −3.50, s.e = 1.17, *t*(6) = −2.99, *p* = 0.037), and hippocampus VBM (*β* = −3.90, s.e = 1.58, *t*(6) = −2.47, *p* = 0.048) only in Tg while WT showed no association. (Fig. 2b).

### VBM is reduced in AD-vulnerable regions in humans

**Fig. 3:**
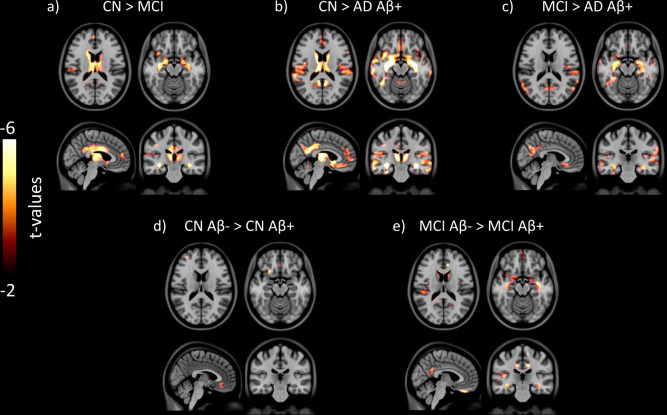
Grey matter density changes in AD spectrum. Significant group contrast maps showing the difference in the VBM between **a** CN and MCI, **b** CN and AD Aβ+, **c** MCI and AD Aβ+, and **d** CN Aβ− and Aβ+ **e** MCI Aβ− and MCI Aβ+. All of the result images are represented on the same *t*-statistic scale.

A voxel-based group contrast on VBM between CN and MCI revealed a significant reduction in the hippocampus, precuneus/posterior cingulate cortex (PCC), medial frontal cortex, and lateral temporal cortex (Fig. 3a and Supplementary Fig. 3 and Table 3). The greater difference was seen in CN and AD contrast where the clusters extended to the orbital frontal cortex, basal lateral temporal cortex (including entorhinal cortex), as well as the parahippocampal gyrus (Fig. 3b and Supplementary Fig. 4 and Table 4). When we compared MCI and AD, the latter showed a significant VBM reduction in precuneus/PCC, medial frontal cortex, basal lateral temporal cortex, inferior parietal cortex, and hippocampus (Fig. 3c and Supplementary Fig. 5 and Table 5). Within the same clinical diagnostic group, CN and MCI showed a difference where CN Aβ+ had a greater VBM reduction in the medial frontal cortex and MCI Aβ+ had greater VBM reduction in the medial frontal cortex, lateral temporal cortex, and right hippocampus compared to the counter Aβ− group, respectively (Fig. 3d, e and Supplementary Figs. 6 and 7 and Tables 6 and 7).

### The NFL concentration and VBM association is modulated by amyloid in AD-vulnerable regions in humans

**Fig. 4:**
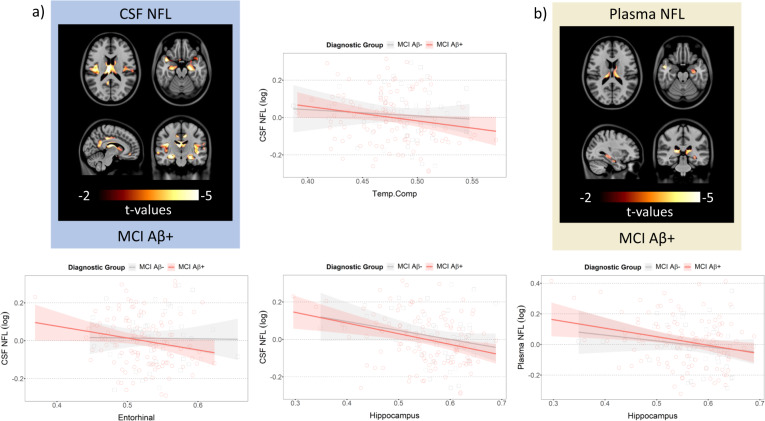
Increased NFL concentrations are associated with reduced grey matter density in MCI Aβ+. **a** Voxel-based maps showing significant associations between CSF NFL concentration and VBM in MCI Aβ+ and ROI-based analyses in MCI Aβ− (in grey) and MCI Aβ+ (in red) showing the association between CSF NFL and Temp.Comp, Entorhinal cortex, or hippocampus. **b** Voxel-based maps showing significant associations between plasma NFL concentration and VBM in MCI Aβ+ and ROI-based analyses in MCI Aβ− (in grey) and MCI Aβ+ (in red) showing the association between plasma NFL and hippocampus.

In MCI Aβ+ cases, increased CSF NFL concentration was significantly associated with reduced VBM in the orbitofrontal cortex, lateral temporal cortex, precuneus/PCC, hippocampus and thalamus based on the voxel-based analysis (Fig. 4a and Supplementary Fig. 8 and Table 8). Also, increased plasma NFL concentration was significantly associated with reduced VBM in the lateral temporal cortex and right hippocampus (*t*(99) = −3.3, *p* = 0.0034) in MCI Aβ+ (Fig. 4b and Supplementary Fig. 9 and Table 9). The ROI-based linear regression analyses revealed significant negative associations between CSF NFL concentration and the Temp.Comp VBM (*β* = −0.93, s.e = 0.39, *t*(104) = −2.38, *p* = 0.018), entorhinal cortex VBM (*β* = −0.85, s.e = 0.32, *t*(104) = −2.51, *p* = 0.015), and both CSF and Plasma NFL were associated with hippocampus VBM (*β* = −0.90, s.e = 0.20, *t*(104) = −4.59, *p* < 0.0001; *β* = −0.70, s.e = 0.24, *t*(99) = −3.03, *p* < 0.013, respectively) in MCI Aβ+ (Fig. 4a, b). In the MCI Aβ+, only the plasma NFL and ROI-VBM analyses showed a significant CSF Aβ_42_:p-tau interaction. Even when this term was included the results stayed the same.

**Fig. 5:**
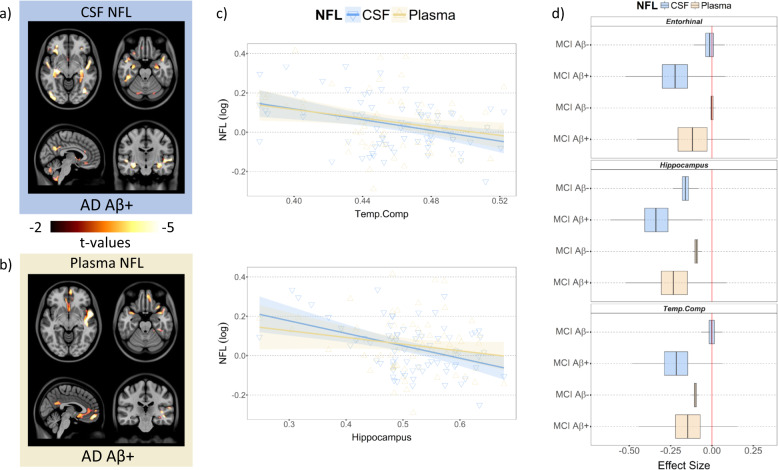
Increased NFL concentrations are associated with reduced grey matter density in AD Aβ+. Voxel-based maps showing significant associations between **a** CSF NFL concentration and VBM, **b** plasma NFL concentration and VBM, and **c** the association between CSF NFL (blue) or plasma NFL (yellow) and Temp.Comp or hippocampus ROIs in AD Aβ+. The shades represent a 95% confidence interval of the regression models. **d** Bootstrap analysis showing the difference in the effect sizes between MCI Aβ+ and Aβ− in CSF or plasma NFL association with VBM.

In AD Aβ+, both CSF and plasma NFL levels were significantly associated with VBM results in dorsal lateral frontal cortex, lateral temporal cortex, precuneus/PCC, medial frontal cortex, angular gyrus, hippocampus, and cerebellar grey in the voxel-based analyses (Fig. 5a, b and Supplementary Figs. 10 and 11 and Tables 10 and 11). Moreover, CSF NFL levels were significantly associated with occipital gyrus VBM measurements in AD Aβ+ (Fig. 5a). The ROI-based linear regression analyses revealed significant negative associations between CSF and the Temp.Comp VBM (*β* = −1.15, s.e = 0.44, *t*(65) = −2.69, *p* = 0.016) and hippocampus VBM (*β* = −0.82, s.e = 0.20, *t*(65) = –3.99, *p* < 0.0001) while plasma NFL and VBM in the Temp.Comp and hippocampus ROIs showed a small trend (*t*(61) = −1.88, *t*(61) = −1.81, respectively, *p* = 0.15).

Although CN Aβ− and Aβ+ and MCI Aβ− groups showed a significant association between NFL and VBM, the RFT-survived clusters were small. CN Aβ− showed a negative association between CSF NFL and lateral front-orbital cortex while CN Aβ+ showed in the cingulate cortex (Supplementary Figs. 12 and 13 and Tables 12 and 13). MCI Aβ− group showed a negative association between plasma NFL and VBM in the insular cortex only (Supplementary Fig. 14 and Table 14). When combining all the groups and adjusting for the diagnosis, only the Aβ+ group showed a negative association between CSF or plasma NFL and precuneus/PCC, basolateral temporal cortex, medial frontal cortex, and striatum (Supplementary Figs. 15 and16 and Tables 15 and 16).

### Greater effect sizes in Aβ+ groups and CSF NFL

To compare the effect size of the association between CSF or plasma NFL and VBM in Aβ− and Aβ+, we applied bootstrapping to run the standardized ROI-based models 2000 times while matching the number of subjects between MCI Aβ− and Aβ+. We selected the MCI group to compare because MCI Aβ+ showed significant results from the ROI analyses. In all three ROIs, MCI Aβ+ showed greater effect size in both CSF and plasma NFL and VBM associations compared to MCI Aβ− (Fig. 5d). This corresponds with our animal results where only the Tg group showed significant associations between CSF NFL and VBM in the homologous ROIs (Fig. 2b). In addition, we applied bootstrapping to run standardized ROI-based models 2000 times in MCI Aβ+ and AD Aβ+. This revealed the association between CSF NFL and VBM had greater effect sizes in most of the regions compared to the association between plasma NFL and VBM (Supplementary Fig. 17).

## Discussion

In this present study, we found an unbiased voxel-based association between NFL concentrations and grey matter density maps in Aβ pathology in humans and animals. NFL has been reported to be a novel neuronal injury or neurodegeneration biomarker but previous studies have only focused on the association between NFL concentrations and a priori ROIs volume over regions typically affected by AD [11, 17, 19]. Assessing the association between NFL concentrations and grey matter changes over the brain allowed us to probe whether the levels of NFL are driven by AD-vulnerable regional neuronal injury or age-related neurodegeneration. Based on our empirical evidence from human and Tg animal model analyses, the Aβ was sufficient to elevate NFL levels and reduce grey matter density and they were associated in AD-vulnerable regions in the presence of Aβ pathology.

Considering that the level of NFL is influenced by other pathophysiological processes (i.e., tau pathology and neuroinflammation) that also are invariably present in clinically manifested AD, it is difficult to fully dissect the degree of contribution from Aβ alone on NFL concentration [8, 10, 18, 37]. Therefore, in this study, we investigated Tg rats with the McGill-R-Thy1-APP model in parallel with the human analyses, using the same methodological approach. Previously, this rat model was shown to have minimal cell death at 18 months of age, reduction in hippocampal volume, and loss of resting-state cingulate connectivity at 16–19 months of age in the presence of Aβ pathology but without tau pathology [25–27]. This most likely reflects the synaptic dysfunction, neuronal injury, and network dysfunction caused by Aβ rather than cell death [26, 27, 38]. Here, our results revealed significant DBM results in the neocortex at 15 months of age and increased CSF NFL concentration in Tg as compared to WT. Therefore, this study strongly supports the notion that the Aβ pathology alone is sufficient to induce neuronal injury with minimal cell death. Association between the Aβ-induced CSF NFL concentrations increase and the DBM measurements reduction was evident in the neocortex and hippocampus where the Aβ pathology is prominent in this animal model. This strongly supports our findings that NFL reflects neuronal injury in AD-related regions in the presence of Aβ pathology.

In addition, our novel findings from the first longitudinal animal CSF analyses revealed that the CSF NFL concentration significantly increased during the healthy aging process as indicated by the results in WT. Also, the CSF Aβ_42/40_ significantly decreased while CSF NFL concentration was significantly increased in Tg animals, showing the inverse relationship between the two biomarkers, thereby replicating the human finding [11]. Considering that the McGill-R-Thy1-APP Tg rat model displays only Aβ pathology without tau pathology, we demonstrated a modest but significant Aβ effect on CSF NFL concentration in Tg compared to WT without the potential effect of tau pathology. Importantly, there was no significant interaction effect between genotype and age. This Tg model has been reported to start accumulating Aβ plaques between the age of 6 and 9 months; the soluble forms of Aβ oligomers dominate prior to this point [25, 26, 39]. Here, we showed that CSF NFL concentration was already elevated before 10 months and remained high throughout the time over, which we collected our samples. This most likely represents the neurotoxic events induced by soluble Aβ oligomers, which in turn increase CSF NFL concentration during the early stage of the pathology in this animal model [38]. Therefore, our study suggests that the association between Aβ and CSF NFL level is established at an early stage of the pathology. Interestingly, it has been reported that there is a significant interaction between the age and genotype on CSF NFL concentration in an APPPS1 mice model [12]. The discrepancy with our results could be due to much more aggressive Aβ pathology accumulation in the APPPS1 mice model as compared to the McGill-R-Thy1-APP rat model, in which the Aβ pathology develops at an equivalent rate as seen in the humans in McGill-R-Thy1-APP rat model [26]. Our longitudinal findings are in agreement with the other recently published longitudinal NFL measurements findings indicating that the rate of serum NFL change increases during the pre-clinical phase of the disease but plateaus before symptoms onset in familial AD, and that CSF NFL levels over time follow a U-shaped curve in sporadic AD [19, 40]. Nonetheless, the animal models of Aβ pathology concurringly argue that Aβ pathology leads to the increased CSF NFL concentration.

Neuronal injury or neurodegeneration is not an AD specific process. Besides AD, increased levels of NFL are also reported in other neurodegenerative conditions such as frontotemporal dementia, progressive supranuclear palsy, and Creutzfeldt-Jakob disease [9, 10, 13, 41, 42]. However, an increasing number of large-scale network analyses have shown a disease-specific vulnerability in an ensemble of regions for each of them [22, 23]. Thus, it is imperative to study if an increased level of a fluid biomarker reflecting neuronal injury or neurodegeneration is linked to anomalies within the network affected by a given disease. Recent evidence based on the association between NFL levels and VBM measurements was in agreement with other studies using a priori defined ROIs in frontotemporal lobar degeneration and Huntington disease, showing that increased NFL concentration was associated with reduced frontal cortex and striatum VBM measurements, respectively. This supports the hypothesis that indeed increased NFL concentrations in those diseases follow neuronal injury in their specific network [10, 13]. To the best of our knowledge, our study is the first to describe voxel-based associations between the NFL levels and VBM measurements in regions targeted specifically by AD. We show that the elevated NFL concentration is associated with reduced VBM measurements in the presence of Aβ pathology in the precuneus/PCC, medial frontal cortex, lateral temporal cortex, inferior parietal cortex, and hippocampus, which are all considered to be AD-vulnerable regions. It is important to note that these cortical regions are also form the default mode network on which AD pathological, functional, and structural abnormalities converge, leading to network failure [21, 22, 43–45]. We dichotomized Aβ pathology based on the previously used cutoff points and included covariates, such as CSF p-tau and/or CSF Aβ_42_:p-Tau interaction that could potentially confound the association between NFL levels and grey matter density maps, and yet, we found correlations only in the Aβ positive groups and in the AD-vulnerable regions [34]. The reason why these regions are vulnerable in AD still remains poorly understood.

There are some important limitations to this study. Due to the modest effect size in the association between NFL concentration and grey matter density maps, the available number of individuals who have both CSF and plasma NFL concentration and MRI data in the ADNI database is overall low, which limited the statistical power of our study. Perhaps this weakness could explain why we observed a minimal association in CN Aβ+. Future studies with a greater number of participants and longitudinal data would be of interest. Another possible reason for the minimal association in CN Aβ+ may be because some CN elderly individuals with high Aβ pathology might actually have minimal levels of toxic soluble Aβ oligomers [46]. Indeed, our animal results suggest that the increase in CSF NFL concentration occurs most likely due to the presence of soluble Aβ oligomers as opposed to that of plaques [38, 39]. In addition, we corrected the analysis for CSF p-tau levels and/or CSF Aβ and p-tau interaction to exclude a potential effect of tau pathology on the association between NFL levels and grey matter density maps. However, it would be interesting to include tau-PET imaging data in a future study as it would allow to control for the different tau loads in different brain regions. Regarding our animal data, although the number of animals in WT and Tg cohorts was not significantly different initially, some of the WT animals developed pituitary tumours during the course of the study. An exploratory analysis revealed that this condition to be a confounding factor due to the significantly increased CSF NFL concentration in the animals that developed those pituitary tumours as compared to the healthy animals (data not included). Therefore, they were excluded in the group comparison analysis.

In summary, our evidence supports that elevated NFL levels in AD results from Aβ-induced neuronal injury in AD-vulnerable regions. NFL is a sensitive biomarker capable of detecting the modest levels of neuronal injury linked to the healthy aging process or to pure Aβ pathology. As such, our study supports the hypothesis that NFL is a novel biomarker of neuronal injury and neurodegeneration that may be utilized within the A/T/N classification scheme and for the evaluation of therapeutic efficacy in clinical trials.

## Supplementary information

Supplementary Tables

Supplementary Figure 1

Supplementary Figure 2

Supplementary Figure 3

Supplementary Figure 4

Supplementary Figure 5

Supplementary Figure 6

Supplementary Figure 7

Supplementary Figure 8

Supplementary Figure 9

Supplementary Figure 10

Supplementary Figure 11

Supplementary Figure 12

Supplementary Figure 13

Supplementary Figure 14

Supplementary Figure 15

Supplementary Figure 16

Supplementary Figure 17

Supplementary Figure Legends
