# Supplementary material for: Amyloid-beta modulates the association between neurofilament light chain and brain atrophy in Alzheimer’s disease
Source: Mol Psychiatry. 2020 Jun 26;26(10):5989–6001. doi: 10.1038/s41380-020-0818-1 (PMC8758474; doi:10.1038/s41380-020-0818-1)
Supplement: Supplementary file 1 — Supplementary Tables [file 41380_2020_818_MOESM1_ESM.docx]

**Title: Amyloid-beta modulates the association between neurofilament light chain and brain atrophy in Alzheimer’s disease.**

**Authors:** Min Su Kang^1,2,3^, Arturo Aliaga Aliaga^1,2,3^, Monica Shin^1,2^, Sulantha Mathotaarachchi^1,2^, Andrea L. Benedet^1,2^, Tharick A. Pascoal^1,2^, Joseph Therriault^1,2^, Mira Chamoun^1,2^, Melissa Savard^1,2^, Gabriel A. Devenyi^2,4^, Axel Mathieu^2^, M. Mallar Chakravarty^2,4,5^, Åsa Sandelius^6^, Kaj Blennow^6,7^, Henrik Zetterberg^6,7,8,9^, Jean-Paul Soucy^3^, A. Claudio Cuello^10^, Gassan Massarweh^3^, Serge Gauthier^1,2,3^, Pedro Rosa-Neto^1,2,3,4^, Alzheimer’s Disease Neuroimaging Initiative^**^

**Affiliation:** Translational Neuroimaging laboratory - McGill University Research Centre for Studying in Aging^1^, Cerebral Imaging Centre – Douglas Research Centre^2^, McConnell Brain Imaging Centre – McGill University^3^, Department of Psychiatry – McGill University^4^, Department of Biomedical Engineering – McGill University^5^, Department of Psychiatry and Neurochemistry, the Sahlgrenska Academy at the University of Gothenburg, Mölndal, Sweden^6^, Clinical Neurochemistry Laboratory, Sahlgrenska University Hospital, Mölndal, Sweden^7^, UK Dementia Research Institute at UCL, London, United Kingdom^8^, Department of Neurodegenerative Disease, UCL Institute of Neurology, Queen Square, London, United Kingdom^9^, Department of Pharmacology and Therapeutics– McGill University^10^

^**^Data used in preparation of this article were obtained from the Alzheimer’s Disease Neuroimaging Initiative database (adni.loni.usc.edu). As such, the investigators within the ADNI contributed to the design and implementation of ADNI and/or provided data but did not participate in analysis or writing of this report. A complete listing of ADNI investigators can be found at: <http://adni.loni.usc.edu/wp-content/uploads/how_to_apply/ADNI_Acknowledgement_List.pdf>

*** Corresponding author**: Pedro Rosa-Neto, MD, PhD.

Translational Neuroimaging Laboratory, McGill University Research Centre for Studies in Aging,

Douglas Hospital, McGill University, Montreal, QC, Canada.

6875 La Salle Blvd - FBC room 3149, Montreal, QC, Canada H4H 1R3.

Email: pedro.rosa@mcgill.ca

**Supplementary Table 1.**

| x | y | z | t-values | Regions | L/R |
| --- | --- | --- | --- | --- | --- |
| 1.904124 | 1.187106 | 5.585437 | 8.18 | Frontal cortex | right |
| 1.165348 | -3.43619 | -7.02465 | 4.479 | Pons | right |
| -3.44943 | 1.553856 | -1.62733 | 3.671 | Striatum | left |
| 1.919853 | -0.88328 | -5.0571 | 3.449 | Thalamus | right |
| -4.93576 | 4.522994 | -3.58051 | 3.423 | Parietotemporal cortex | left |
| -7.33832 | 2.010593 | -4.58955 | 3.095 | Parietotemporal cortex | left |

The standardized stereotaxic coordinates of each peak voxel (> 3 t-value) with minimum 2mm distance apart are displayed with the corresponding t-value and region name from Supplementary Figure 1. All coordinates are in Anterior Commissure origin stereotaxic space.

**Supplementary Table 2.**

| x | y | z | t-values | Regions | L/R |
| --- | --- | --- | --- | --- | --- |
| 2.288319 | 5.160035 | -0.84611 | 13.08 | Parietotemporal cortex | right |
| 2.205505 | 3.090039 | -3.4493 | 7.904 | Hippocampus | right |
| -7.20392 | -0.13745 | -7.78222 | 6.439 | Entorhinal cortex | left |
| 3.159625 | 1.505142 | -0.24537 | 6.041 | Striatum | right |
| 0.69624 | 1.671377 | -15.0027 | 5.881 | Cerebellum | right |
| -2.66543 | -4.06561 | -8.66698 | 5.746 | Pons | left |
| 2.147044 | 1.805813 | 5.929955 | 5.553 | Frontal cortex | right |
| 4.50739 | 0.44114 | -11.7616 | 5.529 | Cerebellum | right |
| 4.464944 | 4.274171 | -1.70373 | 5.516 | Parietotemporal cortex | right |
| -5.72325 | 2.351092 | -3.7788 | 5.428 | Parietotemporal cortex | left |
| 0.600743 | -2.98772 | -7.08551 | 5.148 | Pons | right |
| -4.09508 | 1.41938 | -11.6825 | 4.726 | Cerebellum | left |
| 1.159431 | 2.989946 | -13.5184 | 4.588 | Cerebellum | right |
| 3.452243 | 4.574751 | 4.471624 | 4.523 | Frontal cortex | right |
| -6.8468 | -2.20965 | -5.95076 | 4.363 | Entorhinal cortex | left |
| 7.305979 | -0.48841 | -2.34516 | 4.228 | Parietotemporal cortex | right |
| -4.57095 | -2.48829 | -2.64647 | 3.991 | Entorhinal cortex | left |
| 2.241364 | -1.7763 | 0.471666 | 3.817 | Frontal cortex | right |
| -5.06695 | 1.837376 | -6.32246 | 3.811 | Hippocampus | left |
| -5.65103 | -2.46668 | -11.5227 | 3.674 | Cerebellum | left |
| 0.032244 | -1.03192 | -14.3387 | 3.604 | Cerebellum | right |
| 2.606088 | 0.162798 | -10.1736 | 3.542 | Cerebellum | right |
| -4.04702 | 5.102023 | 1.006788 | 3.49 | Frontal cortex | left |
| 2.208129 | 5.010554 | -4.45162 | 3.473 | Parietotemporal cortex | right |
| 4.073254 | -1.42051 | -12.5859 | 3.305 | Cerebellum | right |
| -1.93241 | 1.372266 | -10.2145 | 3.227 | Cerebellum | left |
| 0.016682 | -1.85438 | -10.4343 | 3.06 | Cerebellum | right |

The standardized stereotaxic coordinates of each peak voxel (> 3 t-value) with minimum 2mm distance apart are displayed with the corresponding t-value and region name from Supplementary Figure 2. All coordinates are in Anterior Commissure origin stereotaxic space.

**Supplementary Table 3.**

| x | y | z | t-values | Regions | L/R |
| --- | --- | --- | --- | --- | --- |
| -20 | -6 | -13 | 5.542 | uncus | left |
| -27 | -39 | 1 | 5.424 | hippocampal formation | left |
| 28 | -39 | -3 | 5.348 | hippocampal formation | right |
| -6 | -21 | 8 | 5.328 | thalamus | left |
| -34 | -28 | -7 | 5.148 | hippocampal formation | left |
| -30 | -12 | -20 | 5.119 | hippocampal formation | left |
| -6 | -3 | 30 | 4.975 | cingulate region | left |
| 36 | -27 | -10 | 4.688 | hippocampal formation | right |
| -9 | -3 | 12 | 4.669 | thalamus | left |
| -12 | 11 | 9 | 4.65 | caudate nucleus | left |
| -40 | -3 | -10 | 4.553 | insula | left |
| -18 | 10 | 19 | 4.532 | caudate nucleus | left |
| 5 | -17 | 4 | 4.515 | thalamus | right |
| 7 | -6 | 13 | 4.39 | thalamus | right |
| 20 | -5 | -14 | 4.363 | uncus | right |
| 26 | 17 | 1 | 4.347 | putamen | right |
| -5 | -27 | 29 | 4.293 | cingulate region | left |
| -40 | -8 | 3 | 4.278 | insula | left |
| 10 | -27 | 6 | 4.271 | thalamus | right |
| 31 | -13 | -19 | 4.269 | hippocampal formation | right |
| -3 | 50 | 12 | 4.166 | cingulate region | left |
| -36 | 16 | -14 | 4.114 | insula | left |
| -4 | -42 | 30 | 4.046 | cingulate region | left |
| 46 | 9 | -3 | 4.012 | inferior frontal gyrus | right |
| -32 | -12 | -43 | 3.96 | inferior temporal gyrus | left |
| -6 | 3 | 3 | 3.956 | thalamus | left |
| -4 | 10 | -4 | 3.901 | caudate nucleus | left |
| 41 | -2 | -9 | 3.899 | insula | right |
| -48 | 2 | 1 | 3.851 | precentral gyrus | left |
| -46 | -22 | 18 | 3.818 | postcentral gyrus | left |
| 32 | 5 | -2 | 3.814 | putamen | right |
| 4 | -27 | 28 | 3.66 | cingulate region | right |
| -21 | -60 | 7 | 3.623 | parahippocampal gyrus | left |
| 36 | 2 | 6 | 3.568 | insula | right |
| -53 | -14 | 13 | 3.514 | postcentral gyrus | left |
| 22 | -65 | -6 | 3.513 | medial occipitotemporal gyrus | right |

The standardized stereotaxic coordinates of each peak voxel (> 3.5 t-value) with minimum 8mm distance apart are displayed with the corresponding t-value and region name from Supplementary Figure 3. All coordinates are in Montreal Neurological Institute (MNI) template space.

**Supplementary Table 4.**

| x | y | z | t-values | Regions | L/R |
| --- | --- | --- | --- | --- | --- |
| -29 | -11 | -18 | 8.831 | hippocampal formation | left |
| -7 | -23 | 8 | 7.271 | thalamus | left |
| -28 | -9 | -38 | 6.863 | inferior temporal gyrus | left |
| -35 | -27 | -9 | 6.836 | hippocampal formation | left |
| 29 | -10 | -17 | 6.589 | hippocampal formation | right |
| 9 | -25 | 8 | 6.425 | thalamus | right |
| 36 | -28 | -11 | 6.388 | hippocampal formation | right |
| -41 | -6 | 2 | 6.023 | insula | left |
| -41 | -5 | -10 | 5.953 | insula | left |
| 12 | -54 | 9 | 5.859 | parahippocampal gyrus | right |
| -4 | 11 | -5 | 5.76 | caudate nucleus | left |
| -32 | -28 | -24 | 5.719 | lateral occipitotemporal gyrus | left |
| -10 | 11 | 7 | 5.537 | caudate nucleus | left |
| -5 | -54 | 20 | 5.446 | precuneus | left |
| -10 | 2 | 13 | 5.413 | caudate nucleus | left |
| 5 | -56 | 20 | 5.399 | precuneus | right |
| -8 | -59 | 31 | 5.386 | precuneus | left |
| -62 | -22 | -6 | 5.28 | superior temporal gyrus | left |
| 42 | -2 | -3 | 5.262 | insula | right |
| -5 | -46 | 32 | 5.246 | cingulate region | left |
| -44 | -55 | -16 | 5.242 | lateral occipitotemporal gyrus | left |
| -56 | -45 | 11 | 5.181 | superior temporal gyrus | left |
| 57 | 3 | 4 | 5.139 | precentral gyrus | right |
| 19 | 1 | -23 | 5.098 | uncus | right |
| -31 | 25 | -2 | 4.957 | insula | left |
| -27 | -50 | -7 | 4.881 | medial occipitotemporal gyrus | left |
| 4 | -44 | 30 | 4.842 | cingulate region | right |
| 26 | -56.5 | -6 | 4.823 | medial occipitotemporal gyrus | right |
| 12 | 13 | 8 | 4.81 | caudate nucleus | right |
| -4 | -34 | 36 | 4.804 | cingulate region | left |
| -14 | -12 | 22 | 4.794 | caudate nucleus | left |
| 27 | 7 | -27 | 4.773 | uncus | right |
| 11 | -61 | 24 | 4.754 | precuneus | right |
| -57 | -3 | -15 | 4.754 | superior temporal gyrus | left |
| 68 | -23 | -5 | 4.709 | middle temporal gyrus | right |
| -9 | -51 | 8 | 4.709 | medial occipitotemporal gyrus | left |
| -50 | -16 | 13 | 4.678 | postcentral gyrus | left |
| -57 | -11 | -32 | 4.667 | inferior temporal gyrus | left |
| -53 | -35 | 25 | 4.603 | supramarginal gyrus | left |
| -43 | -20 | -30 | 4.603 | lateral occipitotemporal gyrus | left |
| 14 | 2 | 17 | 4.517 | caudate nucleus | right |
| 4 | 16 | -8 | 4.516 | cingulate region | right |
| 61 | -30 | 19 | 4.494 | supramarginal gyrus | right |
| 56 | 6 | -22 | 4.486 | superior temporal gyrus | right |
| -62 | -35 | 17 | 4.427 | supramarginal gyrus | left |
| 44 | -15 | -37 | 4.42 | middle temporal gyrus | right |
| -44 | -64 | 6 | 4.397 | middle temporal gyrus | left |
| -63 | -23 | -19 | 4.381 | middle temporal gyrus | left |
| -5 | 8 | -14 | 4.371 | lateral front-orbital gyrus | left |
| -19 | -59 | 4 | 4.369 | parahippocampal gyrus | left |
| -25 | -72.5 | -9 | 4.362 | medial occipitotemporal gyrus | left |
| 50 | -26 | -3 | 4.356 | superior temporal gyrus | right |
| 60 | -25 | -22 | 4.355 | inferior temporal gyrus | right |
| -15 | -48 | 0 | 4.348 | parahippocampal gyrus | left |
| 41 | 14 | -10 | 4.291 | insula | right |
| -34 | 11 | 6 | 4.23 | insula | left |
| -40 | -32 | 38 | 4.204 | postcentral gyrus | left |
| 60 | -16 | -30 | 4.192 | middle temporal gyrus | right |
| -61 | -22 | 11 | 4.185 | superior temporal gyrus | left |
| -26 | -82 | -12 | 4.169 | lingual gyrus | left |
| 44 | 11 | -3 | 4.167 | insula | right |
| 20 | -68 | -7 | 4.149 | medial occipitotemporal gyrus | right |
| 5 | 33 | 20 | 4.128 | cingulate region | right |
| 39 | -21 | 8 | 4.121 | superior temporal gyrus | right |
| 23 | 15 | -2 | 4.096 | putamen | right |
| -28 | -61 | -11 | 4.081 | medial occipitotemporal gyrus | left |
| -31 | 3 | -49 | 4.072 | middle temporal gyrus | left |
| -4 | 28 | -14 | 4.06 | medial front-orbital gyrus | left |
| -57 | -27 | 19 | 4.054 | supramarginal gyrus | left |
| 39 | -2 | 11 | 4.033 | insula | right |
| -49 | -28 | 19 | 4.018 | supramarginal gyrus | left |
| 64 | -42 | 3 | 3.985 | middle temporal gyrus | right |
| 44 | 14 | 29 | 3.954 | middle frontal gyrus | right |
| -47 | 13 | 27 | 3.951 | middle frontal gyrus | left |
| 50 | -28 | 17 | 3.903 | supramarginal gyrus | right |
| 50 | -77 | 1 | 3.898 | inferior occipital gyrus | right |
| 15 | -49 | -22 | 3.892 | cerebellum | right |
| 50 | -16 | -12 | 3.871 | superior temporal gyrus | right |
| -54 | -37 | 0 | 3.858 | middle temporal gyrus | left |
| -45 | 10 | -44 | 3.847 | middle temporal gyrus | left |
| 33 | 25 | 0 | 3.805 | insula | right |
| -5 | 58 | 0 | 3.795 | medial frontal gyrus | left |
| -60 | -40 | -18 | 3.771 | middle temporal gyrus | left |
| -55 | -54 | 0 | 3.746 | middle temporal gyrus | left |
| -38 | -65 | -12 | 3.708 | lateral occipitotemporal gyrus | left |
| -6 | 39 | -14 | 3.707 | medial front-orbital gyrus | left |
| -38 | -11 | -42 | 3.699 | inferior temporal gyrus | left |
| 5 | 29 | -16 | 3.665 | medial front-orbital gyrus | right |
| -6 | 36 | 29 | 3.663 | cingulate region | left |
| 58 | -62 | 11 | 3.635 | middle temporal gyrus | right |
| -65 | -9 | -21 | 3.633 | middle temporal gyrus | left |
| 60 | -60 | -3 | 3.577 | middle temporal gyrus | right |
| 40 | -58 | -10 | 3.546 | lateral occipitotemporal gyrus | right |
| -3 | 46 | 6 | 3.524 | cingulate region | left |
| -2 | 41 | 17 | 3.522 | cingulate region | left |
| 41 | 24 | 2 | 3.508 | inferior frontal gyrus | right |

The standardized stereotaxic coordinates of each peak voxel (> 3.5 t-value) with minimum 8mm distance apart are displayed with the corresponding t-value and region name from Supplementary Figure 4. All coordinates are in Montreal Neurological Institute (MNI) template space.

**Supplementary Table 5.**

| x | y | z | t-values | Regions | L/R |
| --- | --- | --- | --- | --- | --- |
| -26 | -10 | -19 | 5.399 | hippocampal formation | left |
| -29 | -13 | -35 | 5.381 | lateral occipitotemporal gyrus | left |
| -46 | -66 | 7 | 4.886 | middle temporal gyrus | left |
| -45 | -18 | -30 | 4.869 | inferior temporal gyrus | left |
| 25 | -8 | -16 | 4.765 | uncus | right |
| 35 | -28 | -13 | 4.541 | hippocampal formation | right |
| -58 | -11 | -30 | 4.534 | inferior temporal gyrus | left |
| -61 | -19 | -21 | 4.408 | middle temporal gyrus | left |
| 43 | -37 | -20 | 4.34 | lateral occipitotemporal gyrus | right |
| 61 | -28 | 19 | 4.34 | supramarginal gyrus | right |
| 11 | -58 | 9 | 4.251 | parahippocampal gyrus | right |
| -61 | -31 | -3 | 4.231 | superior temporal gyrus | left |
| 25 | -4 | -40 | 4.227 | inferior temporal gyrus | right |
| 7 | 52 | -4 | 4.221 | cingulate region | right |
| 52 | -16 | -12 | 4.221 | middle temporal gyrus | right |
| -20 | -37 | -1 | 4.21 | hippocampal formation | left |
| -34 | -45 | -18 | 4.204 | lateral occipitotemporal gyrus | left |
| 42 | -15 | 4 | 4.19 | superior temporal gyrus | right |
| -30 | -29 | -12 | 4.179 | hippocampal formation | left |
| 58 | 4 | -22 | 4.176 | superior temporal gyrus | right |
| -33 | 22 | 2 | 4.142 | insula | left |
| 23 | -42 | -29 | 4.101 | cerebellum | right |
| 30 | 5 | -23 | 4.092 | uncus | right |
| -33 | -81 | -36 | 4.04 | cerebellum | left |
| -6 | -59 | 32 | 4.024 | precuneus | left |
| 49 | 12 | 25 | 4.014 | inferior frontal gyrus | right |
| -59 | -58 | 18 | 4.009 | middle temporal gyrus | left |
| 54 | -39 | 2 | 3.986 | superior temporal gyrus | right |
| 27 | -12 | -37 | 3.982 | parahippocampal gyrus | right |
| -62 | -6 | -11 | 3.977 | superior temporal gyrus | left |
| 54 | 9 | 9 | 3.929 | precentral gyrus | right |
| -4 | -56 | 23 | 3.915 | precuneus | left |
| 60 | -38 | -20 | 3.893 | inferior temporal gyrus | right |
| -34 | -24 | -28 | 3.892 | lateral occipitotemporal gyrus | left |
| -4 | -65 | 14 | 3.892 | precuneus | left |
| 67 | -31 | 34 | 3.88 | supramarginal gyrus | right |
| -44 | -79 | -12 | 3.8 | lateral occipitotemporal gyrus | left |
| -47 | -56 | -15 | 3.784 | lateral occipitotemporal gyrus | left |
| 65 | -50 | 15 | 3.738 | middle temporal gyrus | right |
| 4 | -55 | 24 | 3.729 | precuneus | right |
| 57 | -65 | 14 | 3.72 | middle temporal gyrus | right |
| 68 | -25 | 27 | 3.714 | supramarginal gyrus | right |
| -48 | 11 | -41 | 3.699 | middle temporal gyrus | left |
| -62 | -14 | -10 | 3.691 | superior temporal gyrus | left |
| -5 | -67 | 34 | 3.689 | precuneus | left |
| 46 | -45 | -23 | 3.687 | lateral occipitotemporal gyrus | right |
| 4 | -64 | 14 | 3.649 | cuneus | right |
| 56 | -63 | 35 | 3.624 | angular gyrus | right |
| -3 | 18 | -8 | 3.618 | cingulate region | left |
| 41 | -3 | -3 | 3.599 | insula | right |
| 62 | -21 | -10 | 3.591 | middle temporal gyrus | right |
| 20 | 9 | -19 | 3.588 | lateral front-orbital gyrus | right |
| 46 | -16 | 17 | 3.584 | supramarginal gyrus | right |
| -38 | 5 | -19 | 3.579 | superior temporal gyrus | left |
| -22 | 9 | -18 | 3.546 | lateral front-orbital gyrus | left |
| -46 | -2 | -47 | 3.527 | inferior temporal gyrus | left |
| 52 | -12 | 13 | 3.517 | postcentral gyrus | right |
| 39 | 15 | 5 | 3.509 | inferior frontal gyrus | right |
| -59 | -5 | 6 | 3.509 | superior temporal gyrus | left |
| -40 | -17 | 0 | 3.506 | insula | left |

The standardized stereotaxic coordinates of each peak voxel (> 3.5 t-value) with minimum 8mm distance apart are displayed with the corresponding t-value and region name from Supplementary Figure 5. All coordinates are in Montreal Neurological Institute (MNI) template space.

**Supplementary Table 6.**

| x | y | z | t-values | Regions | L/R |
| --- | --- | --- | --- | --- | --- |
| -27 | 19 | -13 | 4.644 | lateral front-orbital gyrus | left |
| -31 | 52 | 22 | 3.694 | middle frontal gyrus | left |
| -2 | 31 | -10 | 3.54 | cingulate region | left |

The standardized stereotaxic coordinates of each peak voxel (> 3.5 t-value) with minimum 8mm distance apart are displayed with the corresponding t-value and region name from Supplementary Figure 6. All coordinates are in Montreal Neurological Institute (MNI) template space.

**Supplementary Table 7.**

| x | y | z | t-values | Regions | L/R |
| --- | --- | --- | --- | --- | --- |
| 14 | -46 | 37 | 4.807 | superior parietal lobule | right |
| -6 | 34 | -29 | 4.605 | lateral front-orbital gyrus | left |
| -25 | -47 | 3 | 4.224 | parahippocampal gyrus | left |
| -39 | -7 | 7 | 4.118 | insula | left |
| -20 | 11 | -21 | 4.054 | lateral front-orbital gyrus | left |
| 5 | 29 | -28 | 4.002 | lateral front-orbital gyrus | right |
| -43 | -19 | 8 | 3.97 | superior temporal gyrus | left |
| 38 | -32 | -11 | 3.965 | hippocampal formation | right |
| -10 | -48 | 31 | 3.916 | cingulate region | left |
| -37 | -12 | -19 | 3.902 | hippocampal formation | left |
| 15 | -51 | 5 | 3.766 | parahippocampal gyrus | right |
| 27 | 20 | 43 | 3.68 | middle frontal gyrus | right |
| 5 | 15 | 1 | 3.54 | caudate nucleus | right |

The standardized stereotaxic coordinates of each peak voxel (> 3.5 t-value) with minimum 8mm distance apart are displayed with the corresponding t-value and region name from Supplementary Figure 7. All coordinates are in Montreal Neurological Institute (MNI) template space.

**Supplementary Table 8.**

| x | y | z | t-values | Regions | L/R |
| --- | --- | --- | --- | --- | --- |
| -40 | 10 | -23 | 5.58 | superior temporal gyrus | left |
| -4 | -16 | 15 | 5.452 | thalamus | left |
| 48 | -13 | -2 | 5.186 | superior temporal gyrus | right |
| -42 | -20 | 19 | 4.856 | postcentral gyrus | left |
| 23 | -14 | -21 | 4.811 | hippocampal formation | right |
| -28 | -16 | -13 | 4.61 | hippocampal formation | left |
| 7 | -38 | 35 | 4.372 | cingulate region | right |
| 13 | -17 | 43 | 4.338 | precentral gyrus | right |
| -44 | -8 | 14 | 4.295 | precentral gyrus | left |
| -40 | -4 | 8 | 4.223 | insula | left |
| -29 | -66 | -9 | 4.201 | medial occipitotemporal gyrus | left |
| -31 | -40 | -5 | 4.015 | parahippocampal gyrus | left |
| 40 | -17 | 11 | 4.01 | insula | right |
| 9 | -25 | 38 | 3.997 | cingulate region | right |
| 42 | -6 | 3 | 3.973 | insula | right |
| 4 | 29 | -12 | 3.951 | cingulate region | right |
| -10 | -30 | 38 | 3.911 | cingulate region | left |
| 61 | -11 | 7 | 3.9 | superior temporal gyrus | right |
| -39 | -20 | 3 | 3.846 | superior temporal gyrus | left |
| 8 | 3 | 9 | 3.803 | thalamus | right |
| -44 | 24 | 4 | 3.771 | inferior frontal gyrus | left |
| -36 | 26 | 7 | 3.747 | inferior frontal gyrus | left |
| -63 | -18 | -5 | 3.746 | superior temporal gyrus | left |
| -51 | -24 | -1 | 3.729 | superior temporal gyrus | left |
| -5 | 42 | -11 | 3.719 | cingulate region | left |
| -32 | 26 | -1 | 3.698 | insula | left |
| -46 | -25 | 10 | 3.676 | superior temporal gyrus | left |
| -15 | -50 | 37 | 3.667 | superior parietal lobule | left |
| -6 | -49 | 24 | 3.658 | cingulate region | left |
| -6 | 10 | 1 | 3.641 | caudate nucleus | left |
| 60 | -23 | 11 | 3.628 | superior temporal gyrus | right |
| -32 | -71 | 29 | 3.6 | angular gyrus | left |
| -12 | -18 | 41 | 3.567 | medial frontal gyrus | left |
| -35 | 9 | 8 | 3.517 | insula | left |

The standardized stereotaxic coordinates of each peak voxel (> 3.5 t-value) with minimum 8mm distance apart are displayed with the corresponding t-value and region name from Supplementary Figure 8. All coordinates are in Montreal Neurological Institute (MNI) template space.

**Supplementary Table 9.**

| x | y | z | t-values | Regions | L/R |
| --- | --- | --- | --- | --- | --- |
| -50 | 0 | -21 | 4.551 | superior temporal gyrus | left |
| -14 | -29 | 12 | 4.331 | thalamus | left |
| 11 | -27 | 11 | 4.218 | thalamus | right |
| 4 | -20 | 9 | 4.118 | thalamus | right |

The standardized stereotaxic coordinates of each peak voxel (> 3.5 t-value) with minimum 8mm distance apart are displayed with the corresponding t-value and region name from Supplementary Figure 9. All coordinates are in Montreal Neurological Institute (MNI) template space.

**Supplementary Table 10.**

| x | y | z | t-values | Regions | L/R |
| --- | --- | --- | --- | --- | --- |
| 28 | 17 | -15 | 4.925 | lateral front-orbital gyrus | right |
| -28 | -23 | -11 | 4.923 | hippocampal formation | left |
| 59 | -39 | 31 | 4.824 | angular gyrus | right |
| -37 | -91 | -11 | 4.808 | inferior occipital gyrus | left |
| -23 | 25 | -18 | 4.794 | lateral front-orbital gyrus | left |
| 62 | -30 | 35 | 4.57 | supramarginal gyrus | right |
| -6 | -57 | 28 | 4.498 | precuneus | left |
| -45 | -79 | -9 | 4.493 | lateral occipitotemporal gyrus | left |
| -29 | 41 | -11 | 4.416 | lateral front-orbital gyrus | left |
| 60 | -18 | -2 | 4.392 | superior temporal gyrus | right |
| 1 | 2 | 2 | 4.355 | fornix | left |
| 31 | 39 | -13 | 4.26 | lateral front-orbital gyrus | right |
| -43 | -36 | -22 | 4.225 | lateral occipitotemporal gyrus | left |
| -30 | 27 | -8 | 4.207 | insula | left |
| 34 | -16 | -16 | 4.099 | hippocampal formation | right |
| -16 | -71 | 51 | 4.096 | superior parietal lobule | left |
| -11 | 26 | 33 | 4.05 | medial frontal gyrus | left |
| 20 | -84 | -31 | 4.005 | cerebellum | right |
| 5 | -72 | -35 | 3.989 | cerebellum | right |
| -38 | -28 | -12 | 3.977 | hippocampal formation | left |
| -30 | -9 | -26 | 3.974 | hippocampal formation | left |
| -43 | -82 | 12 | 3.868 | inferior occipital gyrus | left |
| -17 | -82 | -34 | 3.833 | cerebellum | left |
| -49 | 2 | -27 | 3.822 | middle temporal gyrus | left |
| 38 | 26 | 6 | 3.806 | inferior frontal gyrus | right |
| -48 | -45 | -22 | 3.794 | lateral occipitotemporal gyrus | left |
| 61 | -32 | 18 | 3.787 | supramarginal gyrus | right |
| -45 | -76 | 19 | 3.787 | inferior occipital gyrus | left |
| 48 | -42 | 11 | 3.783 | middle temporal gyrus | right |
| 50 | -64 | 33 | 3.781 | angular gyrus | right |
| 46 | -73 | 34 | 3.688 | middle occipital gyrus | right |
| 15 | -65 | 69 | 3.658 | superior parietal lobule | right |
| 46 | 11 | -21 | 3.635 | superior temporal gyrus | right |
| -52 | 11 | -14 | 3.631 | superior temporal gyrus | left |
| 40 | -11 | 0 | 3.615 | insula | right |
| 43 | 1 | -20 | 3.581 | superior temporal gyrus | right |
| -42 | -63 | -43 | 3.576 | cerebellum | left |
| 47 | -70 | 24 | 3.538 | middle occipital gyrus | right |
| -48 | -71 | 5 | 3.515 | inferior occipital gyrus | left |

The standardized stereotaxic coordinates of each peak voxel (> 3.5 t-value) with minimum 8mm distance apart are displayed with the corresponding t-value and region name from Supplementary Figure 10. All coordinates are in Montreal Neurological Institute (MNI) template space.

**Supplementary Table 11.**

| x | y | z | t-values | Regions | L/R |
| --- | --- | --- | --- | --- | --- |
| 58 | 0 | -6 | 5.435 | superior temporal gyrus | right |
| 49 | -10 | -4 | 5.323 | superior temporal gyrus | right |
| 4 | 54 | -14 | 5.288 | medial front-orbital gyrus | right |
| 44 | -8 | -14 | 4.957 | superior temporal gyrus | right |
| 37 | 22 | -17 | 4.791 | insula | right |
| 41 | -10 | -2 | 4.445 | insula | right |
| 6 | 60 | 3 | 4.413 | medial frontal gyrus | right |
| 44 | -18 | 14 | 4.085 | supramarginal gyrus | right |
| -8 | 54 | 17 | 3.93 | medial frontal gyrus | left |
| 12 | 15 | 59 | 3.925 | superior frontal gyrus | right |
| 37 | 7 | -23 | 3.908 | superior temporal gyrus | right |
| -34 | 6 | -21 | 3.869 | superior temporal gyrus | left |
| -3 | -50 | 27 | 3.801 | cingulate region | left |
| 5 | 23 | 56 | 3.758 | medial frontal gyrus | right |
| -7 | 39 | -12 | 3.749 | cingulate region | left |
| 5 | 42 | 1 | 3.72 | cingulate region | right |
| 3 | 15 | -9 | 3.698 | cingulate region | right |
| 60 | -27 | 12 | 3.638 | superior temporal gyrus | right |
| -46 | -57 | -48 | 3.595 | cerebellum | left |
| 3 | -55 | 23 | 3.58 | precuneus | right |
| -4 | 31 | -6 | 3.526 | cingulate region | left |
| -42 | -75 | -31 | 3.514 | cerebellum | left |

The standardized stereotaxic coordinates of each peak voxel (> 3.5 t-value) with minimum 8mm distance apart are displayed with the corresponding t-value and region name from Supplementary Figure 11. All coordinates are in Montreal Neurological Institute (MNI) template space.

**Supplementary Table 12.**

| x | y | z | t-values | Regions | L/R |
| --- | --- | --- | --- | --- | --- |
| -17 | -86 | -41 | 5.023 | cerebellum | left |
| -45 | -64 | -49 | 5.001 | cerebellum | left |
| -26 | 15 | -26 | 4.919 | lateral front-orbital gyrus | left |
| 24 | -83 | -41 | 4.765 | cerebellum | right |
| -34 | -70 | -16 | 4.682 | lateral occipitotemporal gyrus | left |
| -50 | -71 | -3 | 4.606 | inferior occipital gyrus | left |
| 38 | -76 | -44 | 4.605 | cerebellum | right |
| 39 | -79 | -32 | 4.374 | cerebellum | right |
| 26 | -84 | -31 | 4.32 | cerebellum | right |
| 44 | -66 | -51 | 4.219 | cerebellum | right |
| -60 | -57 | -4 | 4.093 | middle temporal gyrus | left |
| -38 | -77 | -31 | 4.057 | cerebellum | left |
| -50 | -5 | 2 | 3.971 | superior temporal gyrus | left |
| -23 | -83 | -27 | 3.676 | cerebellum | left |

The standardized stereotaxic coordinates of each peak voxel (> 3.5 t-value) with minimum 8mm distance apart are displayed with the corresponding t-value and region name from Supplementary Figure 12. All coordinates are in Montreal Neurological Institute (MNI) template space.

**Supplementary Table 13.**

| x | y | z | t-values | Regions | L/R |
| --- | --- | --- | --- | --- | --- |
| 2 | 32 | 10 | 7.357 | cingulate region | right |
| 9 | -87 | -16 | 6.862 | lingual gyrus | right |

The standardized stereotaxic coordinates of each peak voxel (> 3.5 t-value) with minimum 8mm distance apart are displayed with the corresponding t-value and region name from Supplementary Figure 13. All coordinates are in Montreal Neurological Institute (MNI) template space.

**Supplementary Table 14.**

| x | y | z | t-values | Regions | L/R |
| --- | --- | --- | --- | --- | --- |
| 47 | -42 | -18 | 4.077 | inferior temporal gyrus | right |
| -50 | -4 | -1 | 3.965 | superior temporal gyrus | left |
| 50 | -5 | 4 | 3.756 | precentral gyrus | right |
| -51 | -19 | 9 | 3.749 | postcentral gyrus | left |
| -41 | -19 | 16 | 3.656 | postcentral gyrus | left |
| -15 | -7 | -16 | 3.619 | uncus | left |
| 47 | -33 | -21 | 3.531 | lateral occipitotemporal gyrus | right |

The standardized stereotaxic coordinates of each peak voxel (> 3.5 t-value) with minimum 8mm distance apart are displayed with the corresponding t-value and region name from Supplementary Figure 14. All coordinates are in Montreal Neurological Institute (MNI) template space.

**Supplementary Table 15.**

| x | y | z | t-values | Regions | L/R |
| --- | --- | --- | --- | --- | --- |
| 48 | -13 | -3 | 6.768 | superior temporal gyrus | right |
| -29 | -19 | -16 | 6.473 | hippocampal formation | left |
| -41 | 10 | -23 | 6.349 | superior temporal gyrus | left |
| 25 | -13 | -23 | 6.26 | hippocampal formation | right |
| -4 | -15 | 14 | 5.481 | thalamus | left |
| -2 | 11 | -3 | 5.4 | cingulate region | left |
| -33 | -37 | -7 | 5.307 | parahippocampal gyrus | left |
| -40 | -19 | 20 | 5.262 | postcentral gyrus | left |
| -34 | 29 | 2 | 5.049 | inferior frontal gyrus | left |
| 41 | -12 | 1 | 5.045 | insula | right |
| -7 | -54 | 27 | 4.874 | precuneus | left |
| -25 | 6 | -17 | 4.854 | lateral front-orbital gyrus | left |
| -5 | 33 | -15 | 4.831 | medial front-orbital gyrus | left |
| 10 | -23 | 38 | 4.797 | cingulate region | right |
| -43 | -8 | 14 | 4.792 | precentral gyrus | left |
| 9 | -11 | 41 | 4.705 | medial frontal gyrus | right |
| -33 | 15 | 8 | 4.704 | insula | left |
| 6 | 10 | 0 | 4.695 | caudate nucleus | right |
| -11 | 21 | 34 | 4.618 | cingulate region | left |
| -13 | -36 | 40 | 4.608 | cingulate region | left |
| -23 | 34 | -18 | 4.581 | lateral front-orbital gyrus | left |
| 31 | -39 | -8 | 4.575 | parahippocampal gyrus | right |
| -30 | 24 | -7 | 4.563 | insula | left |
| -43 | 22 | 4 | 4.534 | inferior frontal gyrus | left |
| 52 | 21 | 17 | 4.522 | inferior frontal gyrus | right |
| -8 | -56 | 14 | 4.521 | precuneus | left |
| 41 | -17 | 9 | 4.503 | insula | right |
| -40 | -4 | 6 | 4.484 | insula | left |
| 10 | 42 | 8 | 4.414 | cingulate region | right |
| -45.5 | 9.5 | 4 | 4.41 | inferior frontal gyrus | left |
| -7 | 7 | 2 | 4.389 | caudate nucleus | left |
| 3 | 2 | 4 | 4.376 | thalamus | right |
| 4 | 29 | -13 | 4.362 | medial front-orbital gyrus | right |
| 59 | -47 | 23 | 4.352 | angular gyrus | right |
| 45 | 11 | -20 | 4.299 | superior temporal gyrus | right |
| 62 | -12 | 7 | 4.289 | superior temporal gyrus | right |
| 30 | 17 | -15 | 4.282 | insula | right |
| 10 | -53 | 28 | 4.241 | precuneus | right |
| -9 | 29 | 24 | 4.222 | cingulate region | left |
| 9 | 43 | -8 | 4.205 | medial front-orbital gyrus | right |
| 62 | -32 | 34 | 4.205 | angular gyrus | right |
| -39 | -21 | 3 | 4.193 | superior temporal gyrus | left |
| -51 | -11 | 10 | 4.192 | postcentral gyrus | left |
| -64 | -18 | -5 | 4.103 | superior temporal gyrus | left |
| 35 | -35 | -18 | 4.025 | lateral occipitotemporal gyrus | right |
| 4 | -14 | 14 | 4.004 | thalamus | right |
| 50 | 26 | 4 | 4.001 | inferior frontal gyrus | right |
| 40 | 26 | 4 | 3.999 | inferior frontal gyrus | right |
| 6 | -38 | 36 | 3.998 | cingulate region | right |
| 35 | 38 | -13 | 3.981 | lateral front-orbital gyrus | right |
| 61 | -17 | -2 | 3.968 | superior temporal gyrus | right |
| -53 | 14 | -15 | 3.956 | superior temporal gyrus | left |
| -52 | -38 | 8 | 3.942 | superior temporal gyrus | left |
| 13 | -57 | 12 | 3.924 | parahippocampal gyrus | right |
| -9 | -11 | 41 | 3.909 | medial frontal gyrus | left |
| 49 | -17 | 16 | 3.907 | supramarginal gyrus | right |
| 42 | -8 | 14 | 3.797 | precentral gyrus | right |
| -62 | -48 | -20 | 3.792 | inferior temporal gyrus | left |
| -42 | -31 | 20 | 3.766 | supramarginal gyrus | left |
| 68 | -40 | 18 | 3.757 | superior temporal gyrus | right |
| -33 | -26 | 15 | 3.75 | insula | left |
| 33 | 20 | 8 | 3.73 | insula | right |
| 24 | 33 | -17 | 3.719 | lateral front-orbital gyrus | right |
| 28 | 4 | -21 | 3.679 | uncus | right |
| 13 | 34 | 22 | 3.678 | cingulate region | right |
| 37 | 6 | 10 | 3.666 | precentral gyrus | right |
| 57 | -29 | 22 | 3.653 | supramarginal gyrus | right |
| 13 | 27 | 30 | 3.633 | medial frontal gyrus | right |
| -18 | -5 | -12 | 3.625 | uncus | left |
| 27 | -59 | -5 | 3.579 | medial occipitotemporal gyrus | right |
| 49 | -70 | 29 | 3.579 | middle occipital gyrus | right |
| -47 | -24 | 10 | 3.572 | superior temporal gyrus | left |
| 6 | 45 | -26 | 3.566 | lateral front-orbital gyrus | right |
| 24 | -8 | -35 | 3.542 | parahippocampal gyrus | right |
| 46 | -63 | -41 | 3.531 | cerebellum | right |
| 45 | -28 | 15 | 3.51 | superior temporal gyrus | right |
| 55 | 13 | 9 | 3.505 | inferior frontal gyrus | right |

The standardized stereotaxic coordinates of each peak voxel (> 3.5 t-value) with minimum 8mm distance apart are displayed with the corresponding t-value and region name from Supplementary Figure 15. All coordinates are in Montreal Neurological Institute (MNI) template space.

**Supplementary Table 16.**

| x | y | z | t-values | Regions | L/R |
| --- | --- | --- | --- | --- | --- |
| 7 | 60 | 15 | 4.481 | medial frontal gyrus | right |
| 7 | 44 | 31 | 4.201 | medial frontal gyrus | right |
| 64 | -11 | 15 | 4.199 | postcentral gyrus | right |
| -38 | 10 | -22 | 4.166 | superior temporal gyrus | left |
| 47 | -15 | -3 | 4.123 | superior temporal gyrus | right |
| 35 | -15 | -20 | 4.022 | hippocampal formation | right |
| 9 | 52 | 23 | 3.993 | medial frontal gyrus | right |
| -28 | 16 | -20 | 3.959 | lateral front-orbital gyrus | left |
| 54 | -5 | -7 | 3.955 | superior temporal gyrus | right |
| 24 | -10 | -24 | 3.912 | hippocampal formation | right |
| 5 | 54 | -14 | 3.816 | medial front-orbital gyrus | right |
| 34 | -25 | -13 | 3.758 | hippocampal formation | right |
| 33 | 9 | -25 | 3.634 | uncus | right |
| 39 | -14 | -1 | 3.566 | insula | right |
| 32 | -33 | -10 | 3.537 | hippocampal formation | right |
| -55 | 16 | -16 | 3.53 | superior temporal gyrus | left |
| 10 | -31 | 5 | 3.507 | thalamus | right |

The standardized stereotaxic coordinates of each peak voxel (> 3.5 t-value) with minimum 8mm distance apart are displayed with the corresponding t-value and region name from Supplementary Figure 16. All coordinates are in Montreal Neurological Institute (MNI) template space.
