# Supplementary figures and images for: Amyloid-beta modulates the association between neurofilament light chain and brain atrophy in Alzheimer’s disease
Source: Mol Psychiatry. 2020 Jun 26;26(10):5989–6001. doi: 10.1038/s41380-020-0818-1 (PMC8758474; doi:10.1038/s41380-020-0818-1)

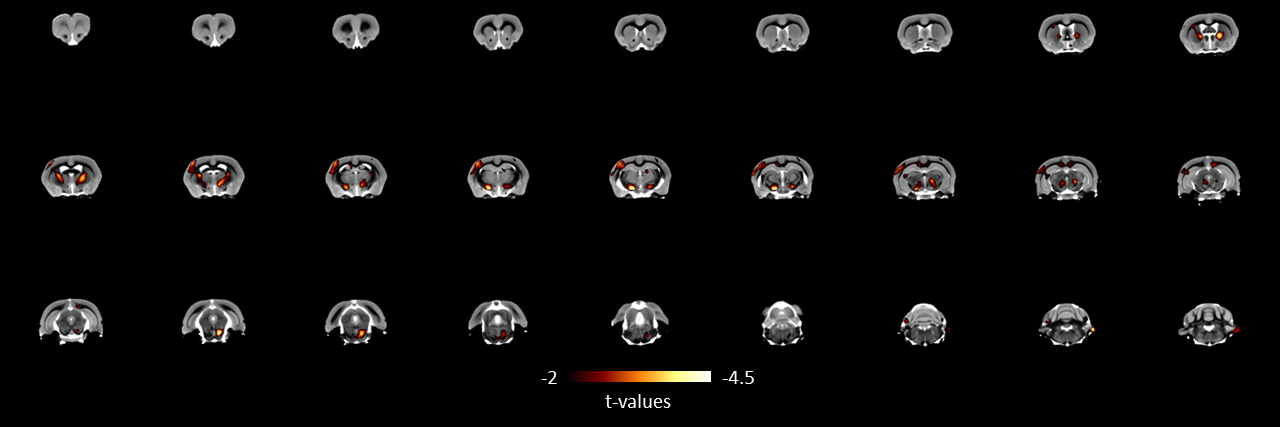

Supplement: Supplementary file 2 — Supplementary Figure 1 [file 41380_2020_818_MOESM2_ESM.tif]

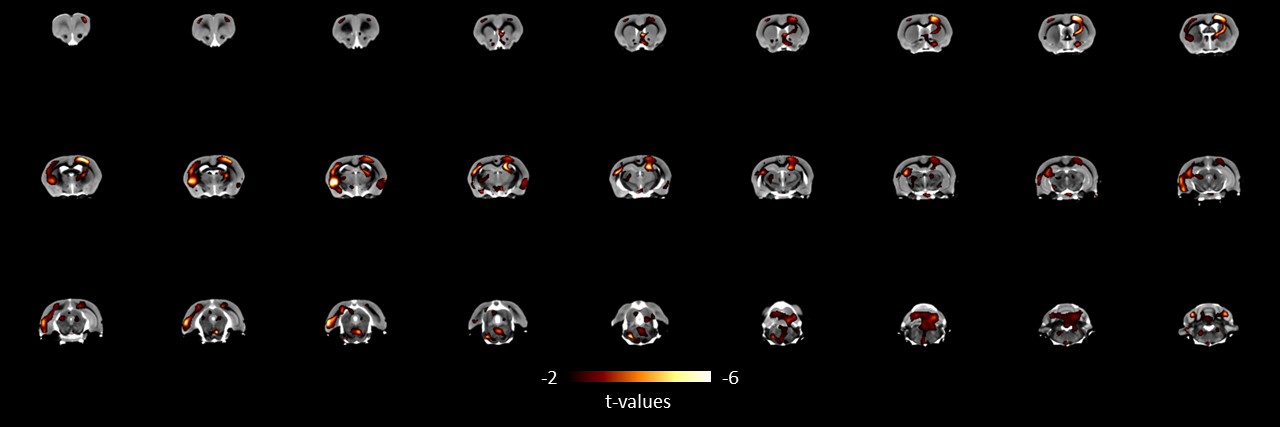

Supplement: Supplementary file 3 — Supplementary Figure 2 [file 41380_2020_818_MOESM3_ESM.tif]

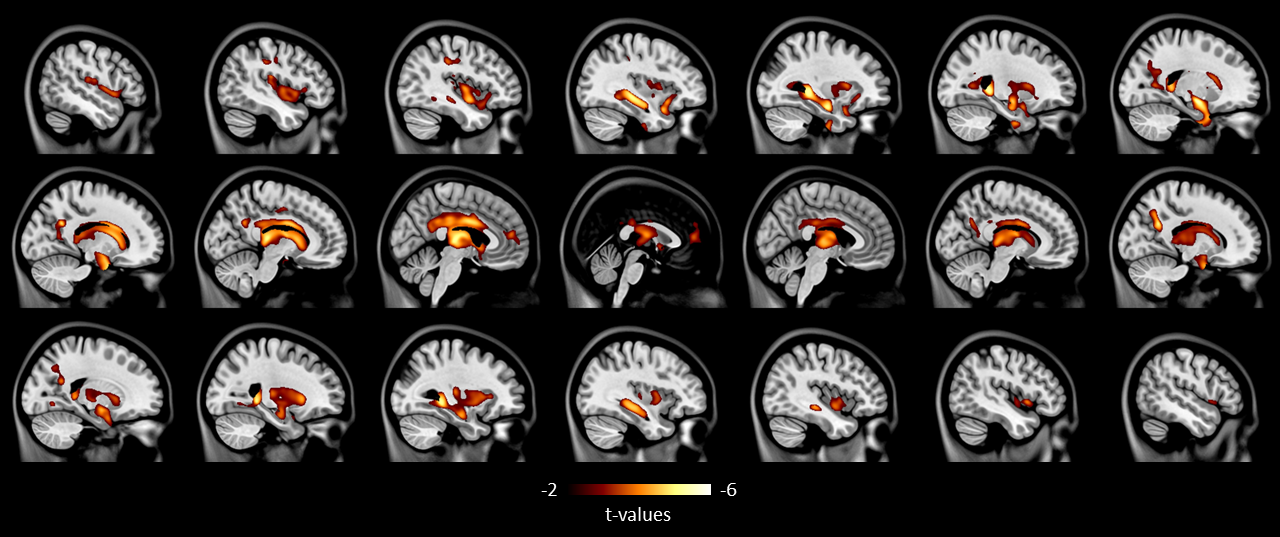

Supplement: Supplementary file 4 — Supplementary Figure 3 [file 41380_2020_818_MOESM4_ESM.tif]

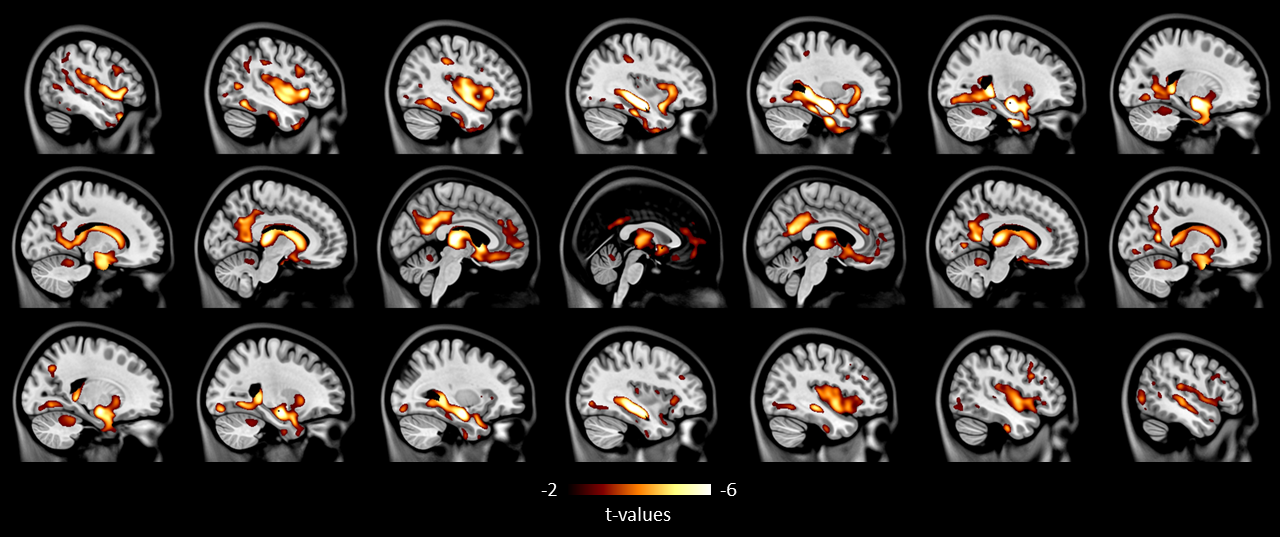

Supplement: Supplementary file 5 — Supplementary Figure 4 [file 41380_2020_818_MOESM5_ESM.tif]

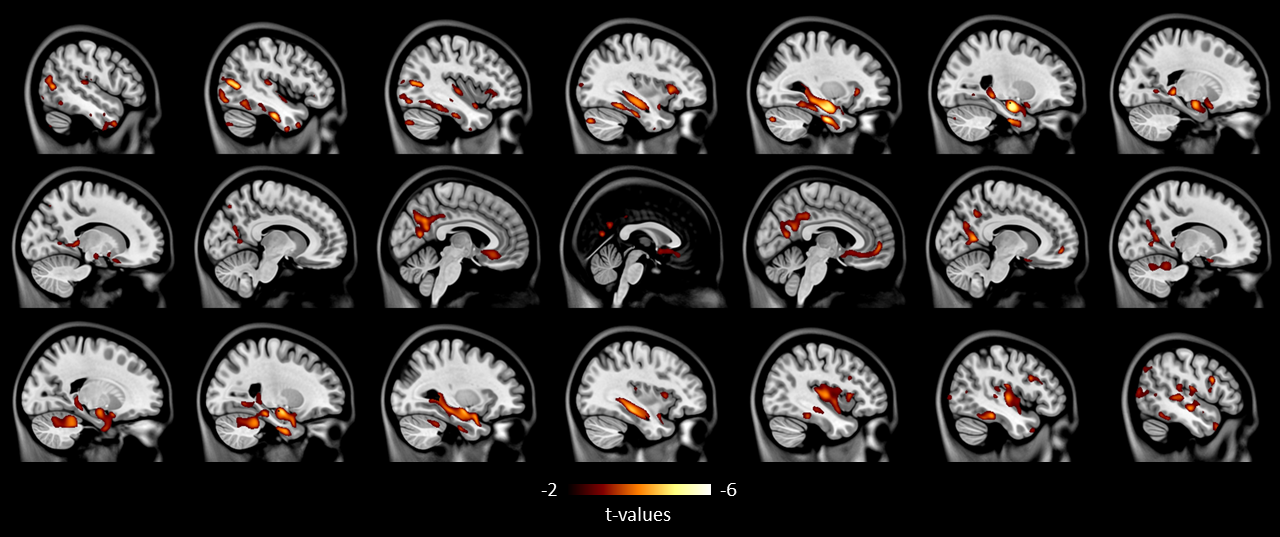

Supplement: Supplementary file 6 — Supplementary Figure 5 [file 41380_2020_818_MOESM6_ESM.tif]

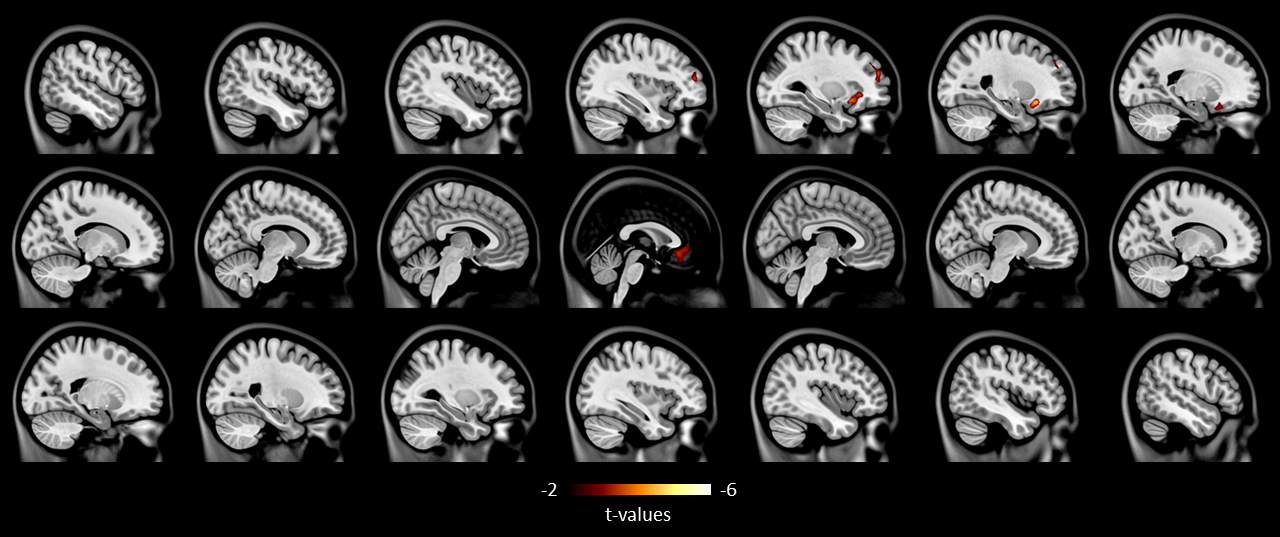

Supplement: Supplementary file 7 — Supplementary Figure 6 [file 41380_2020_818_MOESM7_ESM.tif]

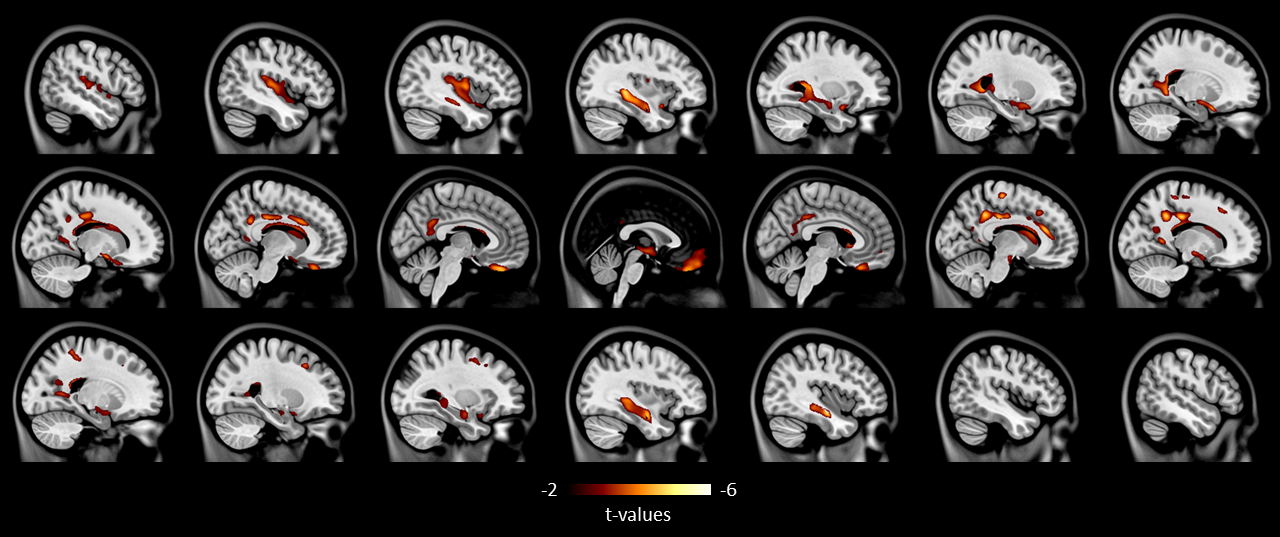

Supplement: Supplementary file 8 — Supplementary Figure 7 [file 41380_2020_818_MOESM8_ESM.tif]

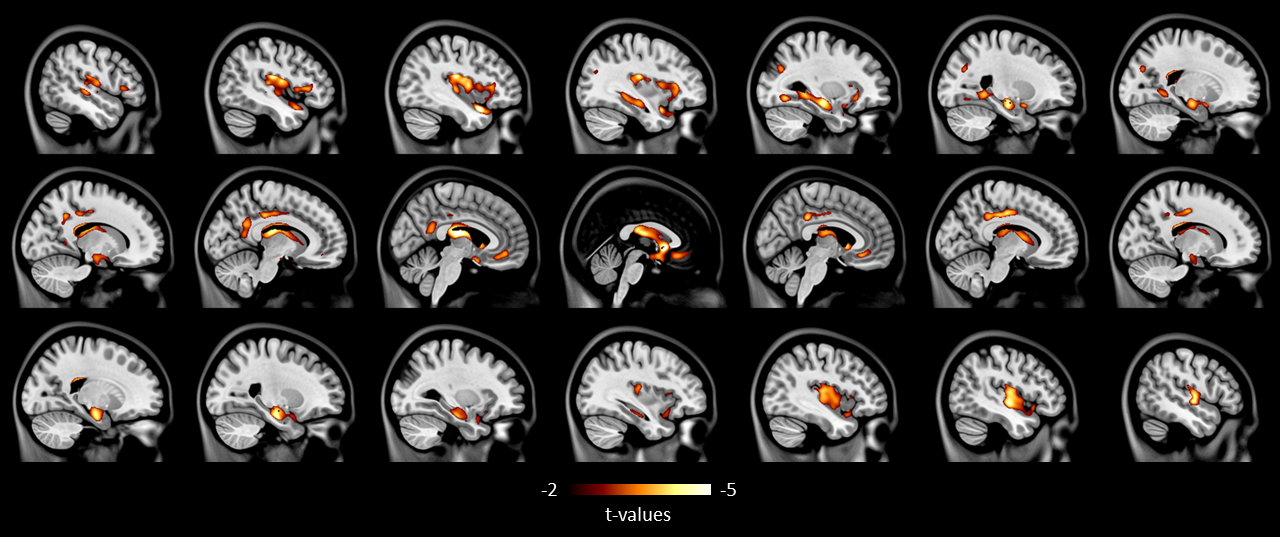

Supplement: Supplementary file 9 — Supplementary Figure 8 [file 41380_2020_818_MOESM9_ESM.tif]

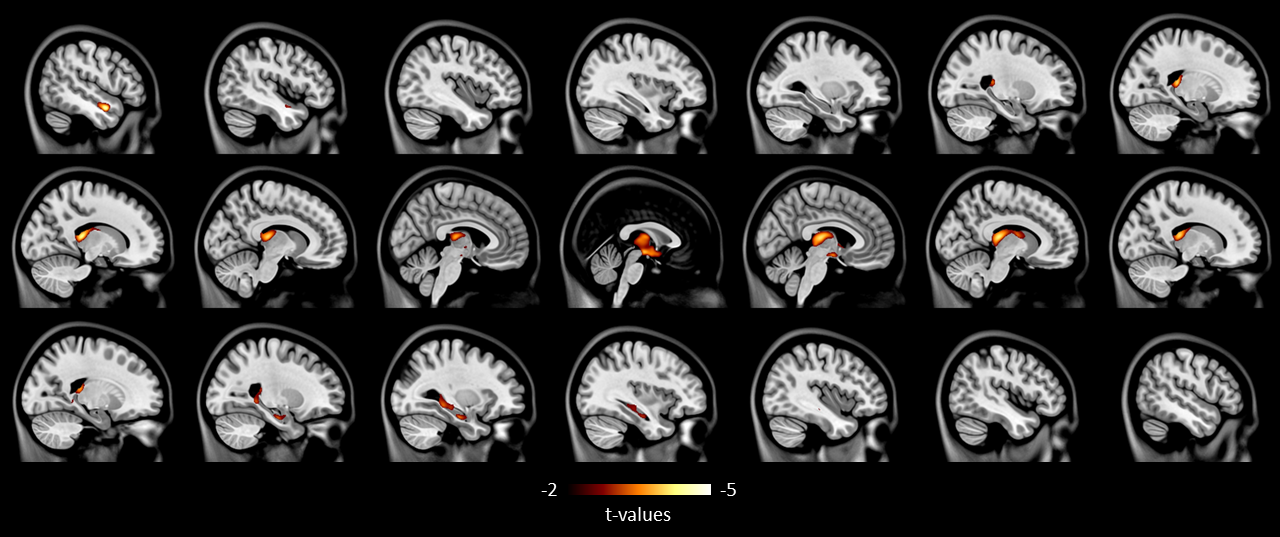

Supplement: Supplementary file 10 — Supplementary Figure 9 [file 41380_2020_818_MOESM10_ESM.tif]

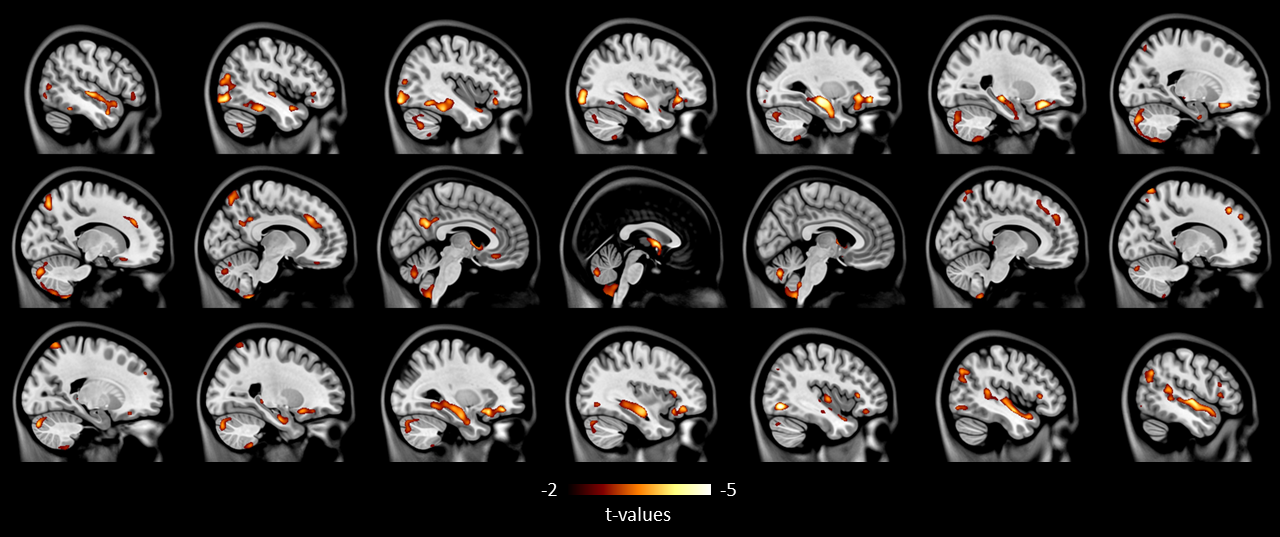

Supplement: Supplementary file 11 — Supplementary Figure 10 [file 41380_2020_818_MOESM11_ESM.tif]

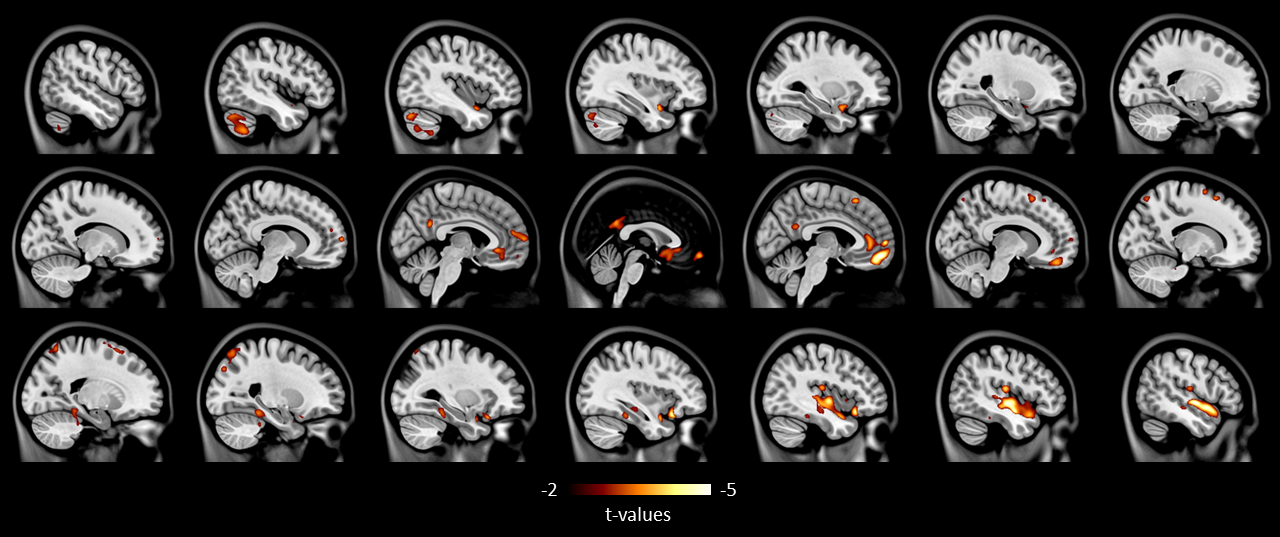

Supplement: Supplementary file 12 — Supplementary Figure 11 [file 41380_2020_818_MOESM12_ESM.tif]

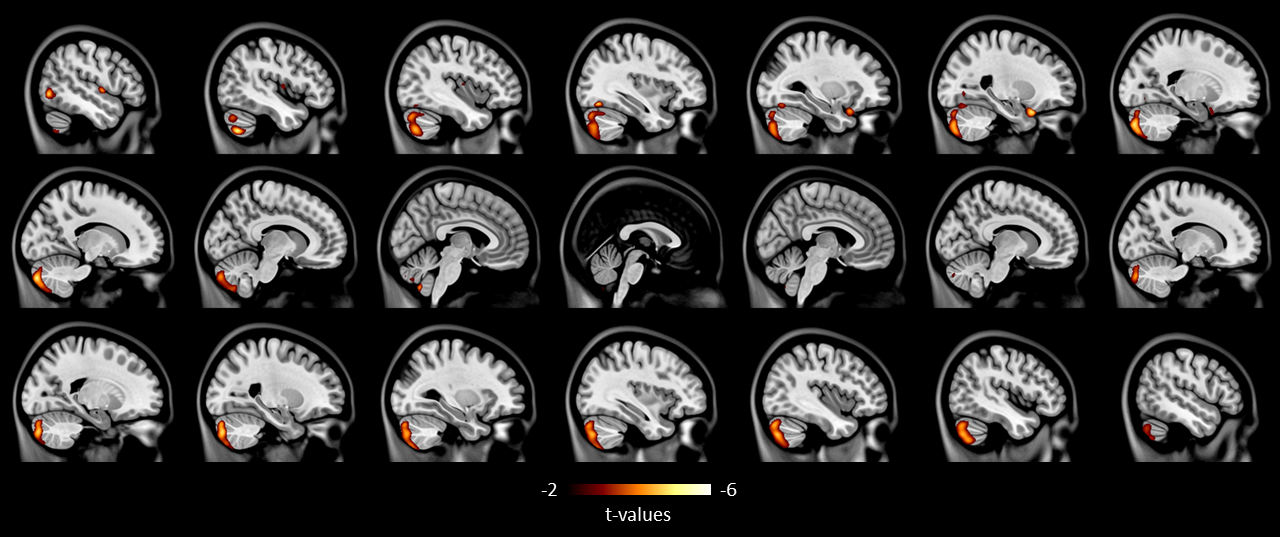

Supplement: Supplementary file 13 — Supplementary Figure 12 [file 41380_2020_818_MOESM13_ESM.tif]

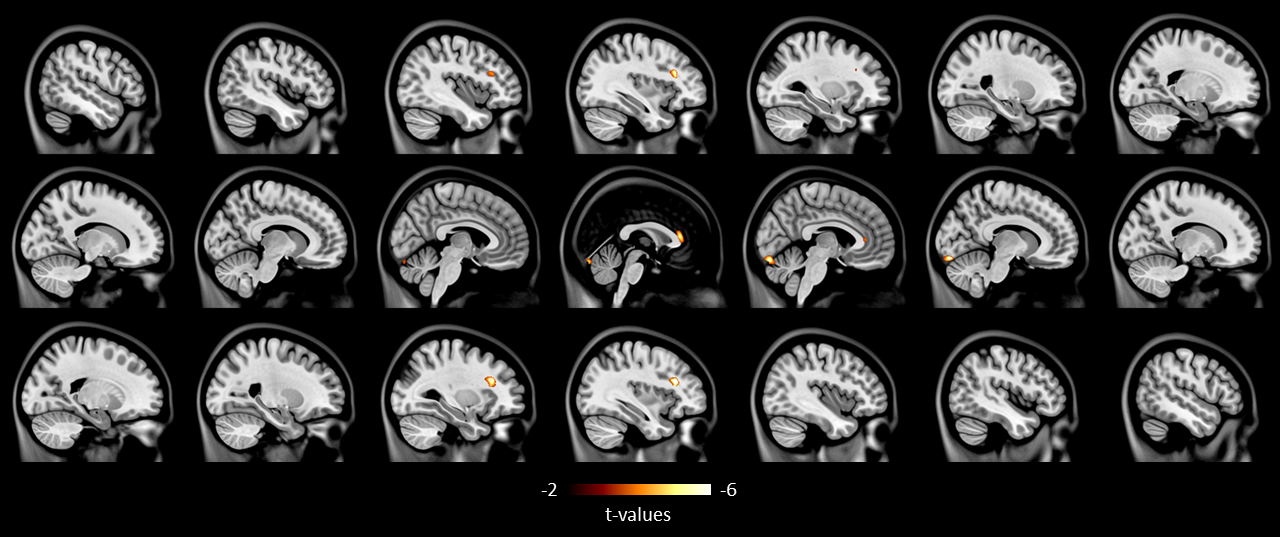

Supplement: Supplementary file 14 — Supplementary Figure 13 [file 41380_2020_818_MOESM14_ESM.tif]

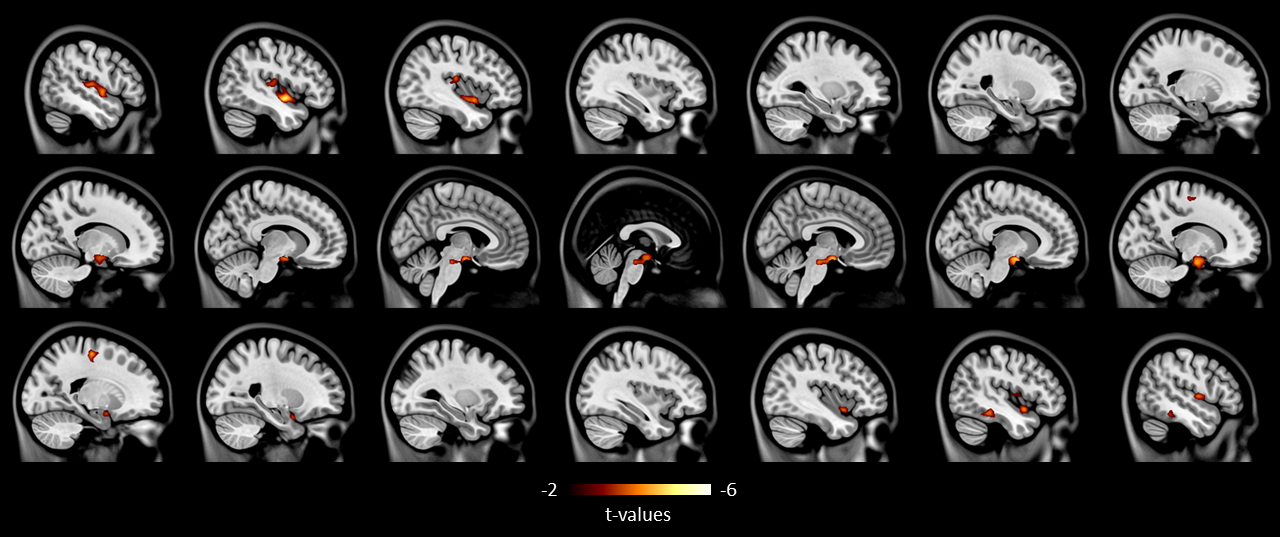

Supplement: Supplementary file 15 — Supplementary Figure 14 [file 41380_2020_818_MOESM15_ESM.tif]

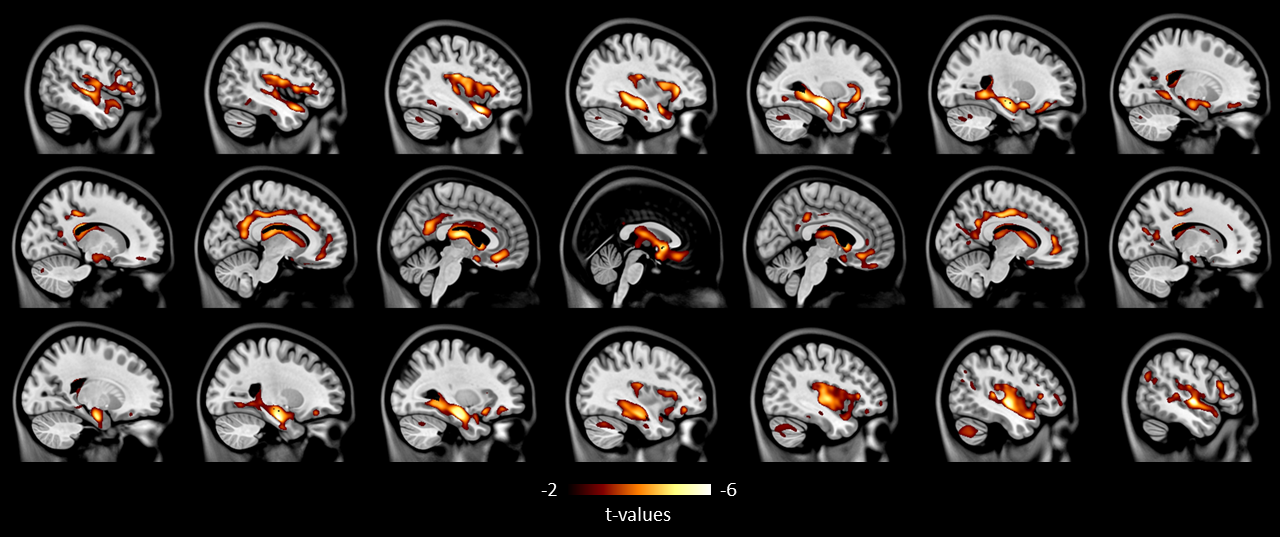

Supplement: Supplementary file 16 — Supplementary Figure 15 [file 41380_2020_818_MOESM16_ESM.tif]

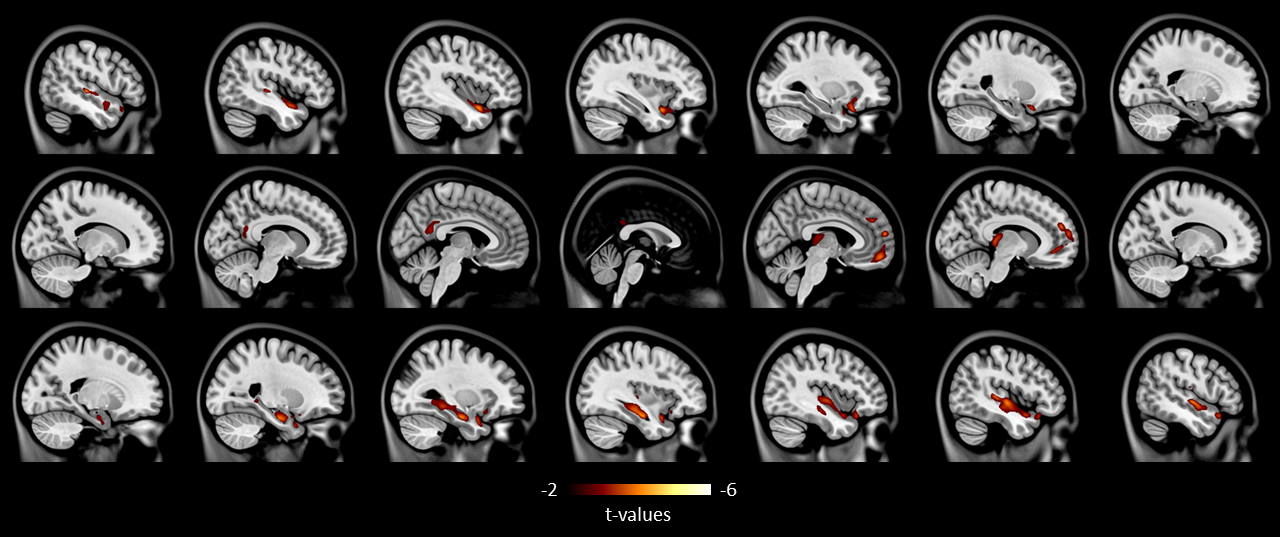

Supplement: Supplementary file 17 — Supplementary Figure 16 [file 41380_2020_818_MOESM17_ESM.tif]

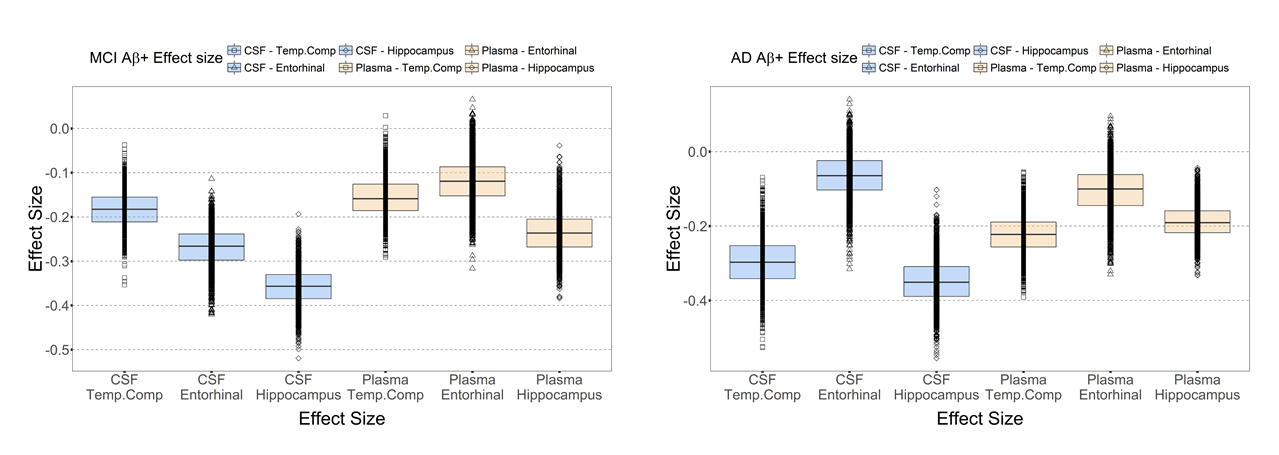

Supplement: Supplementary file 18 — Supplementary Figure 17 [file 41380_2020_818_MOESM18_ESM.tif]
