# Supplementary material for: Amyloid-beta modulates the association between neurofilament light chain and brain atrophy in Alzheimer’s disease
Source: Mol Psychiatry. 2020 Jun 26;26(10):5989–6001. doi: 10.1038/s41380-020-0818-1 (PMC8758474; doi:10.1038/s41380-020-0818-1)
Supplement: Supplementary file 19 — Supplementary Figure Legends [file 41380_2020_818_MOESM19_ESM.docx]

**Title: Amyloid-beta modulates the association between neurofilament light chain and brain atrophy in Alzheimer’s disease.**

**Authors:** Min Su Kang^1,2,3^, Arturo Aliaga Aliaga^1,2,3^, Monica Shin^1,2^, Sulantha Mathotaarachchi^1,2^, Andrea L. Benedet^1,2^, Tharick A. Pascoal^1,2^, Joseph Therriault^1,2^, Mira Chamoun^1,2^, Melissa Savard^1,2^, Gabriel A. Devenyi^2,4^, Axel Mathieu^2^, M. Mallar Chakravarty^2,4,5^, Åsa Sandelius^6^, Kaj Blennow^6,7^, Henrik Zetterberg^6,7,8,9^, Jean-Paul Soucy^3^, A. Claudio Cuello^10^, Gassan Massarweh^3^, Serge Gauthier^1,2,3^, Pedro Rosa-Neto^1,2,3,4^, Alzheimer’s Disease Neuroimaging Initiative^**^

**Affiliation:** Translational Neuroimaging laboratory - McGill University Research Centre for Studying in Aging^1^, Cerebral Imaging Centre – Douglas Research Centre^2^, McConnell Brain Imaging Centre – McGill University^3^, Department of Psychiatry – McGill University^4^, Department of Biomedical Engineering – McGill University^5^, Department of Psychiatry and Neurochemistry, the Sahlgrenska Academy at the University of Gothenburg, Mölndal, Sweden^6^, Clinical Neurochemistry Laboratory, Sahlgrenska University Hospital, Mölndal, Sweden^7^, UK Dementia Research Institute at UCL, London, United Kingdom^8^, Department of Neurodegenerative Disease, UCL Institute of Neurology, Queen Square, London, United Kingdom^9^, Department of Pharmacology and Therapeutics– McGill University^10^

^**^Data used in preparation of this article were obtained from the Alzheimer’s Disease Neuroimaging Initiative database (adni.loni.usc.edu). As such, the investigators within the ADNI contributed to the design and implementation of ADNI and/or provided data but did not participate in analysis or writing of this report. A complete listing of ADNI investigators can be found at: <http://adni.loni.usc.edu/wp-content/uploads/how_to_apply/ADNI_Acknowledgement_List.pdf>

*** Corresponding author**: Pedro Rosa-Neto, MD, PhD.

Translational Neuroimaging Laboratory, McGill University Research Centre for Studies in Aging,

Douglas Hospital, McGill University, Montreal, QC, Canada.

6875 La Salle Blvd - FBC room 3149, Montreal, QC, Canada H4H 1R3.

Email: pedro.rosa@mcgill.ca

**Supplementary Figure 1.**

A voxel-based group contrast between WT vs Tg at 15 months with RFT correction. The standardized stereotaxic coordinates of each peak voxel (> 3 t-value) with minimum 2mm distance apart are reported in the Supplementary Table 1.

**Supplementary Figure 2.**

A voxel-based association between CSF NFL concentrations and DBM in Tg at 15 months with RFT correction. The standardized stereotaxic coordinates of each peak voxel (> 3 t-value) with minimum 2mm distance apart are reported in the Supplementary Table 2.

**Supplementary Figure 3.**

A voxel-based group contrast between CN and MCI with RFT correction. The standardized stereotaxic coordinates of each peak voxel (> 3.5 t-value) with minimum 8mm distance apart are reported in the Supplementary Table 3.

**Supplementary Figure 4.**

A voxel-based group contrast between CN and AD with RFT correction. The standardized stereotaxic coordinates of each peak voxel (> 3.5 t-value) with minimum 8mm distance apart are reported in the Supplementary Table 4.

**Supplementary Figure 5.**

A voxel-based group contrast between MCI and AD with RFT correction. The standardized stereotaxic coordinates of each peak voxel (> 3.5 t-value) with minimum 8mm distance apart are reported in the Supplementary Table 5.

**Supplementary Figure 6.**

A voxel-based group contrast between CN Aβ- and Aβ+ with RFT correction. The standardized stereotaxic coordinates of each peak voxel (> 3.5 t-value) with minimum 8mm distance apart are reported in the Supplementary Table 6.

**Supplementary Figure 7.**

A voxel-based group contrast between MCI Aβ- and Aβ+ with RFT correction. The standardized stereotaxic coordinates of each peak voxel (> 3.5 t-value) with minimum 8mm distance apart are reported in the Supplementary Table 7.

**Supplementary Figure 8.**

A voxel-based association between CSF NFL concentrations and VBM in MCI Aβ+ with RFT correction. The standardized stereotaxic coordinates of each peak voxel (> 3.5 t-value) with minimum 8mm distance apart are reported in the Supplementary Table 8.

**Supplementary Figure 9.**

A voxel-based association between plasma NFL concentrations and VBM in MCI Aβ+ with RFT correction. The standardized stereotaxic coordinates of each peak voxel (> 3.5 t-value) with minimum 8mm distance apart are reported in the Supplementary Table 9.

**Supplementary Figure 10.**

A voxel-based association between CSF NFL concentrations and VBM in AD Aβ+ with RFT correction. The standardized stereotaxic coordinates of each peak voxel (> 3.5 t-value) with minimum 8mm distance apart are reported in the Supplementary Table 10.

**Supplementary Figure 11.**

A voxel-based association between plasma NFL concentrations and VBM in AD Aβ+ with RFT correction. The standardized stereotaxic coordinates of each peak voxel (> 3.5 t-value) with minimum 8mm distance apart are reported in the Supplementary Table 11.

**Supplementary Figure 12.**

A voxel-based association between CSF NFL concentrations and VBM in CN Aβ- with RFT correction. The standardized stereotaxic coordinates of each peak voxel (> 3.5 t-value) with minimum 8mm distance apart are reported in the Supplementary Table 12.

**Supplementary Figure 13.**

A voxel-based association between CSF NFL concentrations and VBM in CN Aβ+ with RFT correction. The standardized stereotaxic coordinates of each peak voxel (> 3.5 t-value) with minimum 8mm distance apart are reported in the Supplementary Table 13.

**Supplementary Figure 14.**

A voxel-based association between plasma NFL concentrations and VBM in MCI Aβ- with RFT correction. The standardized stereotaxic coordinates of each peak voxel (> 3.5 t-value) with minimum 8mm distance apart are reported in the Supplementary Table 14.

**Supplementary Figure 15.**

A voxel-based association between CSF NFL concentrations and VBM in all Aβ+ combined with RFT correction. The standardized stereotaxic coordinates of each peak voxel (> 3.5 t-value) with minimum 8mm distance apart are reported in the Supplementary Table 15.

**Supplementary Figure 16.**

A voxel-based association between CSF NFL concentrations and VBM in all Aβ+ combined with RFT correction. The standardized stereotaxic coordinates of each peak voxel (> 3.5 t-value) with minimum 8mm distance apart are reported in the Supplementary Table 16.

**Supplementary Figure 17.**

2000 bootstrapped standardized effect sizes comparison in MCI Aβ+ or AD Aβ+. Following Bonferroni multiple comparison correction, all the contrasts were significantly different (p < 0.0001) from each other in MCI Aβ+ or AD Aβ+.
